# Efficacy and Safety of Atrasentan in Patients with IgA Nephropathy Receiving Sodium-Glucose Cotransporter 2 Inhibitors: Placebo-Controlled, Crossover Trial

**DOI:** 10.1681/ASN.0000001076

**Published:** 2026-03-29

**Authors:** Hiddo J.L. Heerspink, Irene L. Noronha, Jose Luis Górriz, Soo Kun Lim, Sradha S. Kotwal, Gianna M. Kirsztajn, José Barros Neto, Jessica Ryan, Mei Sian Fu, Sung-Gyun Kim, Jonathan Barratt, Yasmin Brahmbhatt, Greggory J. Housler, Rong Jiao, Marion Dahlke, Amit Lodha, Amy K. Mottl

**Affiliations:** 1 The George Institute for Global Health, University of New South Wales, Sydney, New South Wales, Australia; 2 Department of Clinical Pharmacy and Pharmacology, University Medical Center Groningen, University of Groningen, Groningen, The Netherlands; 3 Renal Division, University of São Paulo Medical School, São Paulo, Brazil; 4 Hospital Clínico Universitario, INCLIVA Biomedical Research Institute, Valencia, Spain; 5 Universidad de Valencia, Valencia, Spain; 6 Department of Medicine, University of Malaya, Kuala Lumpur, Malaysia; 7 Department of Nephrology, Prince of Wales Hospital, Sydney, New South Wales, Australia; 8 Universidade Federal de São Paulo, São Paulo, Brazil; 9 Clinical Nephrology Department of the Brazilian Society of Nephrology, São Paulo, Brazil; 10 Monash Health, Melbourne, Victoria, Australia; 11 Hospital Sultanah Aminah, Johor Bahru, Malaysia; 12 Hallym University Sacred Heart Hospital, Anyang, Republic of Korea; 13 The Mayer IgA Nephropathy Laboratories, University of Leicester, Leicester, United Kingdom; 14 The John Walls Renal Unit, Leicester General Hospital, Leicester, United Kingdom; 15 Novartis Pharmaceuticals Corporation, East Hanover, New Jersey; 16 Novartis Pharma AG, Basel, Switzerland; 17 University of North Carolina School of Medicine, Chapel Hill, North Carolina

**Keywords:** CKD, clinical nephrology, clinical trial, glomerular disease, GN, IgA nephropathy, kidney disease, proteinuria, SGLT2 inhibitors

## Abstract

**Key Points:**

- Patients with IgA nephropathy and proteinuria are at risk of kidney failure despite use of guideline-recommended renin-angiotensin system and sodium-glucose cotransporter 2 (SGLT2) inhibition.
- Atrasentan reduces proteinuria in IgA nephropathy, but its efficacy as adjunct to renin-angiotensin system and SGLT2 inhibition has not been rigorously tested.
- Atrasentan provided a clinically meaningful reduction in proteinuria in adults with IgA nephropathy treated with renin-angiotensin system and SGLT2 inhibitors.

**Background:**

Atrasentan, a highly selective endothelin-A receptor antagonist, is approved for proteinuria reduction in adults with IgA nephropathy. Renin-angiotensin system inhibitors (RASi) and sodium-glucose cotransporter 2 inhibitors (SGLT2i) are guideline recommended, yet the additional benefit of atrasentan has not been rigorously determined.

**Methods:**

We performed a randomized, double-blind, placebo-controlled crossover study of atrasentan in adults with IgA nephropathy, eGFR ≥30 ml/min per 1.73 m^2^, and urinary protein >0.5 g/d while on maximal, stable RASi and SGLT2i. Participants were randomized 1:1 to either sequence AB or sequence BA (0.75 mg atrasentan [A] once daily during period 1 and matching placebo [B] during period 2, or vice versa), with a 12-week washout period in between. The primary end point was the change in urinary protein-to-creatinine ratio (UPCR) to week 12. The secondary end point was the change in UPCR to week 24. Safety end points included the type, incidence, severity, seriousness, and relatedness of adverse events (AEs).

**Results:**

We recruited 54 participants with mean age 48 years (SD 12), 43% female, mean (SD) eGFR 63 (22) ml/min per 1.73 m^2^, and median UPCR 1.0 g/g (Q1–Q3, 0.7–1.4). Treatment with atrasentan versus placebo resulted in a difference in geometric mean percentage change in UPCR at week 12 of −25.3% (95% confidence interval, −36.8 to −11.7; *P <* 0.001). The treatment difference in UPCR between atrasentan versus placebo during treatment period 2 at week 24 was −26.4% (95% confidence interval, −45.8 to −0.0). There was one unrelated serious adverse event. Fluid retention events were uncommon, and none required hospitalization. There were no study drug discontinuations due to treatment-related adverse events and no deaths.

**Conclusions:**

Atrasentan provided a clinically meaningful reduction in proteinuria in adults with IgA nephropathy and proteinuria ≥0.5 g/d treated with RASi and SGLT2i therapy. Atrasentan was well tolerated, and no new safety signals emerged.

**Clinical Trial registry name and registration number:**

NCT05834738.

## Visual Abstract

**Figure:**
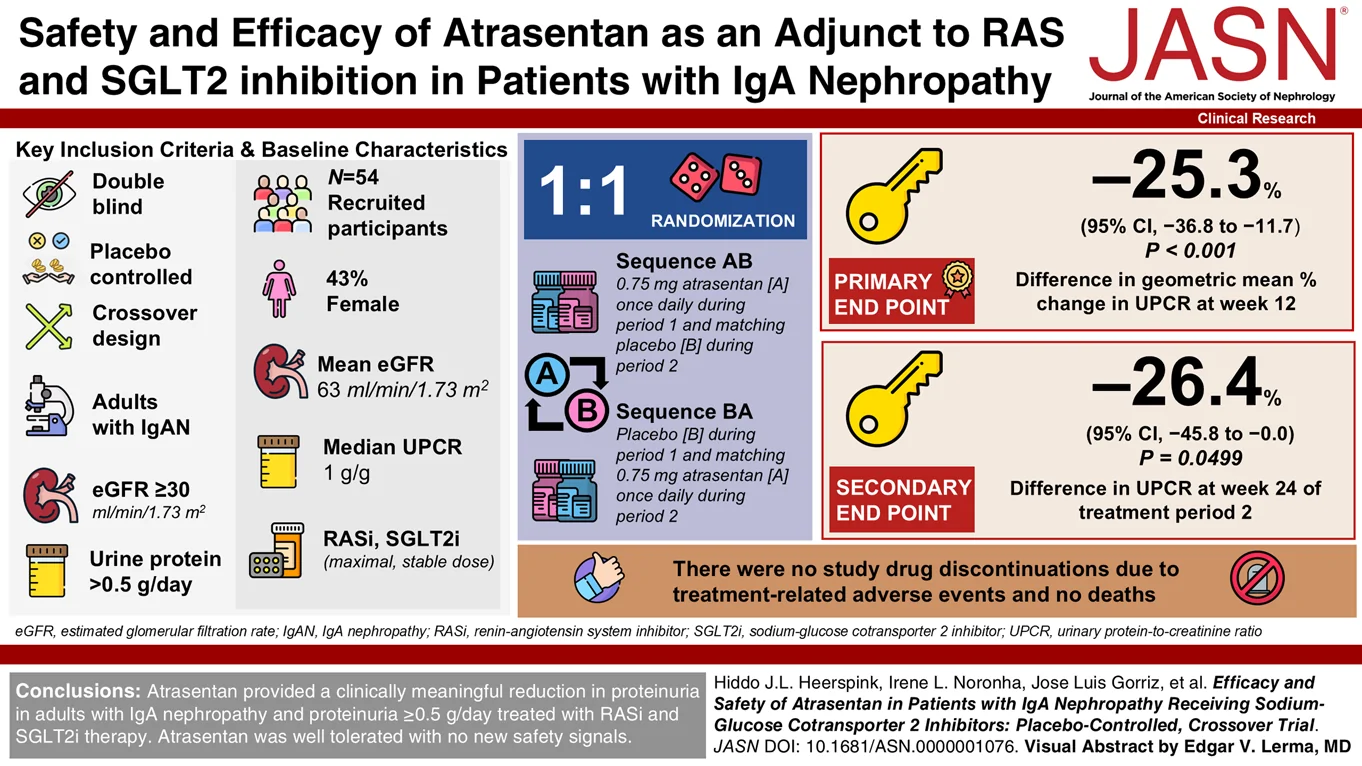

## Introduction

IgA nephropathy is the most common primary GN worldwide, with up to 50% of patients progressing to kidney failure within 10-20 years.^1,2^ Emerging data show that patients with IgA nephropathy and proteinuria ≥0.5 g/d are at risk of progressive kidney function loss.^3^ Accordingly, the Kidney Disease Improving Global Outcomes guideline for the management of IgA nephropathy suggests aiming for strict proteinuria control, with a goal of <0.5 g/d, ideally <0.3 g/d, and a stable eGFR; combination treatment strategies are likely necessary to achieve this goal.^3^ Renin-angiotensin system (RAS) inhibition and sodium-glucose cotransporter 2 (SGLT2) inhibitors are guideline-recommended therapies to slow eGFR decline in patients with IgA nephropathy.^3^ However, despite optimized RAS inhibition and SGLT2 inhibitor use, residual proteinuria over 0.5 g/d remains common, indicating that an unmet need persists.^2^ The development of several new effective and safe drug classes, including endothelin receptor antagonists (ERAs), raises the possibility to combine existing and emerging treatments to achieve the Kidney Disease Improving Global Outcomes guideline-recommended proteinuria treatment goal in order to halt progression of IgA nephropathy.

Endothelin-1 is a potent vasoconstrictor implicated in the pathophysiology of IgA nephropathy.^4^ Binding of endothelin-1 to the endothelin type A (ETA) receptor causes endothelial dysfunction, podocyte damage, extracellular matrix deposition, tubular interstitial fibrosis, kidney inflammation, and proteinuria.^5^ Atrasentan is a potent and highly selective ETA receptor antagonist.^6^ Atrasentan reduced proteinuria by 36% compared with placebo in patients with IgA nephropathy at risk of CKD progression concurrently treated with a maximum tolerated dose of RAS inhibitors in the Atrasentan in patients with IgA Nephropathy (ALIGN) trial.^7^ These effects were present regardless of the severity of proteinuria or degree of kidney impairment.^7^ These results led to the accelerated approval of atrasentan to reduce proteinuria in adults with IgA nephropathy at risk of rapid disease progression in the United States.^8^

The evolving treatment landscape and increasing use of SGLT2 inhibitors in patients with IgA nephropathy warrants the design of clinical trials to examine the efficacy and safety of adding ERAs as an adjunct to SGLT2 and RAS inhibition. Adding ERAs to SGLT2 inhibitors is a pharmacologically sensible strategy as fluid retention, a possible side effect of ERAs, may be mitigated by the diuretic properties of SGLT2 inhibitors.^5,9^ *Post hoc* analysis of the Study of Diabetic Nephropathy with Atrasentan trial, in patients with type 2 diabetes and CKD, showed that 6 weeks of treatment with combined SGLT2 inhibitor/atrasentan versus atrasentan alone decreased body weight, a surrogate for fluid retention, and further decreased albuminuria.^10^

To more robustly assess the effects of atrasentan when given in combination with standard of care including RAS and SGLT2 inhibitors, we designed the Atrasentan and Sodium-Glucose Cotransporter 2 Inhibitor Efficacy and Safety (ASSIST) trial, a randomized, double-blind, placebo-controlled crossover study in patients with IgA nephropathy. We hypothesized that atrasentan compared with placebo on a background of SGLT2 and RAS inhibitors would be efficacious in reducing proteinuria.

## Methods

### Study Design and Participants

The ASSIST trial was a randomized, double-blind, placebo-controlled crossover trial designed to evaluate the effects of the selective ETA receptor antagonist atrasentan as an adjunct to SGLT2 and RAS inhibitors in adults with IgA nephropathy and increased proteinuria. The study was conducted at 30 sites in six countries. Eligible participants were adults 18 years or older with biopsy-proven IgA nephropathy and an eGFR ≥30 ml/min per 1.73 m^2^. Participants were required to receive a stable maximum tolerated dose of angiotensin-converting enzyme inhibitor or angiotensin receptor blocker for at least 12 weeks before study entry.

Participants receiving treatment with an SGLT2 inhibitor at a stable dose for at least 8 weeks before study entry were required to have a total urinary protein excretion of >0.5 g/d. Participants who were not using stable doses of SGLT2 inhibitors for at least 8 weeks entered a run-in period of 8 weeks during which they received a SGLT2 inhibitor at a stable dose. The choice of the SGLT2 inhibitor was left to the discretion of the site investigator. These participants were required to have a total urinary protein excretion of at least 0.85 g/d at screening and at least 0.5 g/d at the end of the 8-week SGLT2 inhibitor run-in period.

Main exclusion criteria included concurrent causes of CKD other than IgA nephropathy; nephrotic syndrome; type 1 diabetes; use of systemic immunosuppressants including systemic corticosteroids; known history of heart failure or prior hospital admissions for conditions relating to fluid overload including, but not limited to, pulmonary edema; B-type natriuretic peptide (BNP) >200 pg/ml; and hemoglobin <9 g/dl. The full list of inclusion and exclusion criteria is described in the Supplemental Material.

All participants provided written informed consent before any study-specific procedures. The trial protocol was approved by national competent authorities and institutional ethics committees of all participating centers and complied with the Declaration of Helsinki and Good Clinical Practice guidelines. The study was registered at ClinicalTrials.gov (NCT05834738).

### Intervention and Randomization

**Figure 1:**
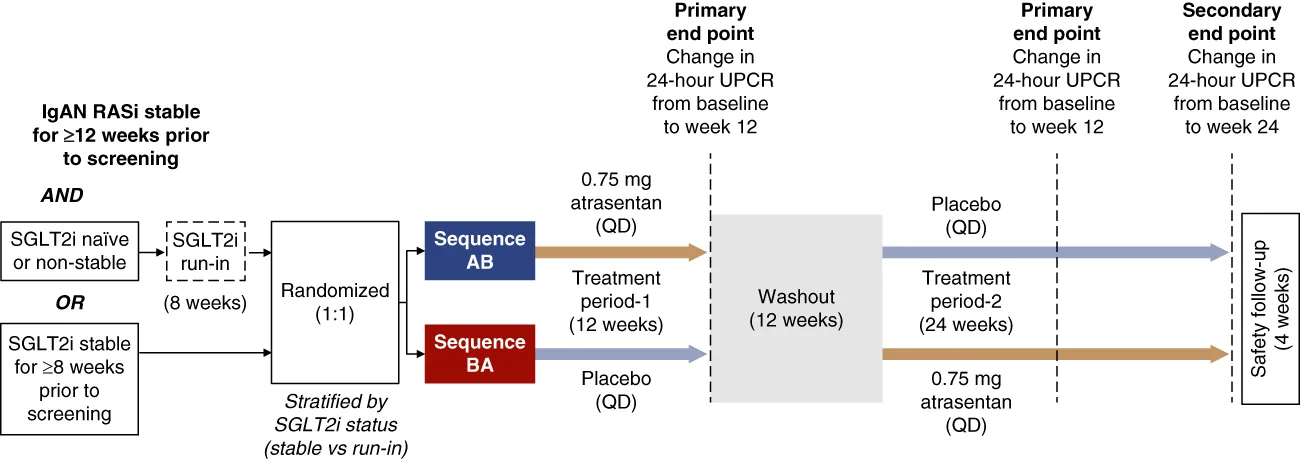
**Study design.** IgA nephropathy, immunoglobulin A nephropathy; QD, once daily; RASi, renin-angiotensin system inhibitor; SGLT2i, sodium-glucose cotransporter 2 inhibitor; UPCR, urinary protein-to-creatinine ratio. Figure reprinted with permission from ref. 11.

Participants underwent a crossover intervention consisting of two consecutive treatment periods separated by a 12-week washout period. The duration of treatment period 1 was 12 weeks, and treatment period 2 was 24 weeks. In treatment period 2, 24 weeks were included to observe the efficacy of proteinuria reduction beyond 12 weeks. Treatment consisted of atrasentan 0.75 mg (A) once daily or matched placebo (B) (Figure 1). Participants were randomized 1:1 to either sequence AB or sequence BA (in which they received 0.75 mg atrasentan once daily during period 1 and matching placebo during period 2, or vice versa), with a 12-week washout period in between. Randomization was stratified by stable versus run-in SGLT2 inhibitor use. Randomization was performed using an interactive response technology system (that generated the random allocation sequence and assigned treatment intervention to participants) supplied by the sponsor, Novartis. Investigators, participants, and sponsor staff remained blinded to treatment allocation throughout the trial. Placebo tablets had the same appearance and odor as atrasentan tablets to ensure blinding to study treatment allocation. Principal investigators were responsible for enrolling the participants.

### Procedures and Measurements

After randomization, in-person study assessments were performed at day 1; weeks 2, 6, and 12 during treatment period 1; and at day 1 and weeks 2, 6, 12, and 24 during treatment period 2. Phone visits occurred at weeks 1, 4, and 8, and additionally in treatment period 2, weeks 16 and 20. During the 12-week washout period, an in-person visit was performed at week 6, while phone visits occurred at weeks 4, 8, and 12. At each in-person visit, vital signs, body weight, and adverse events (AEs) were recorded. BP measurements were conducted with the participant in a semisupine position. Blood samples were drawn at each visit and analyzed at central certified laboratories for hematologic and biochemical assessments. Twenty-four–hour urine collections were performed at the start (day 1) and week 12 of each treatment period and week 24 in treatment period 2. Aliquots from 24-hour urinary specimens were sent for total protein, albumin, and creatinine assessments.

### End Points

The primary end point was the change from baseline in urinary protein-to-creatinine ratio (UPCR) after 12 weeks of atrasentan 0.75 mg/d versus placebo. The secondary end point was the change from baseline in UPCR after 24 weeks' treatment in period 2. An exploratory end point was the change in eGFR from baseline to 24 weeks during period 2. Safety end points included the type, incidence, severity, seriousness, and relatedness of treatment-emergent adverse events (TEAEs); TEAEs of special interest (TEAESIs) included events of fluid retention, hypotension, and anemia. Safety laboratory markers included hematology, including hemoglobin, and complete chemistry including liver enzymes, urinalysis, and BNP.

### Statistical Analyses

#### Power and Sample Size

A target of approximately 52 participants was planned for analysis to ensure a clinically meaningful difference could be detected between atrasentan and placebo in the primary end point of proteinuria reduction (UPCR) at week 12 in addition to the secondary end point of 24-week UPCR change from baseline in treatment period 2. Approximately 52 participants (approximately 26 per treatment sequence) were estimated to provide 83% power using a two-sided pairwise test to detect a treatment effect of at least 0.288 in natural log-transformed UPCR (equivalent to a clinically meaningful reduction in UPCR of 25%)^12^ from baseline to week 12 between atrasentan and placebo at the 5% significance level, assuming a SD of 0.67% and 5% rate of early withdrawal from the study before week 12 in treatment period 2. Using the same assumptions as described above and 5% rate of early withdrawal before week 24 in treatment period 2, 52 participants were estimated to provide approximately 80% power using a two-sided two-sample *t* test to detect a treatment effect of at least 0.56 in natural log transformed UPCR (43% reduction) over 24 weeks in study treatment period 2.

### Statistical Methods

The analysis of the primary efficacy end point was performed using a mixed model that included the change from baseline of natural log UPCR at week 12 within each treatment period as an outcome. The model also included the fixed effects of sequence, period, and treatment; a random effect of participants nested within sequences; and covariates of period-specific baseline natural log UPCR as a continuous variable and the randomization stratification factor of SGLT2 inhibitor status (stable versus run-in). Three supplementary prespecified sensitivity analyses with different strategies to handle intercurrent events and missing end points were proposed to assess robustness (Supplemental Table 1).

The analysis of the secondary efficacy end point was performed using a mixed model for repeated measures, which included the change in natural log UPCR from the baseline of treatment period 2 to each postbaseline measurement through week 24 as the outcome. The model also included the fixed effects of treatment, visit, and treatment-by-visit interaction, with covariates of treatment period 2 baseline natural log UPCR and the randomization stratification factor of SGLT2 inhibitor status (stable versus run-in). The covariance structure was assumed to be unstructured and the same in each treatment sequence.

The analysis of the exploratory end point was performed on eGFR change from the baseline of treatment period 2 to each postbaseline measurement through week 24 using a mixed model for repeated measures as the outcome. The model also included the fixed effects of treatment, visit, and treatment-by-visit interaction, with covariates of treatment period 2 baseline natural log UPCR and eGFR as continuous variables and the randomization stratification factor of SGLT2 inhibitor status (stable versus run-in). The covariance structure was assumed to be unstructured and the same in each treatment sequence.

Safety outcomes were summarized by treatment period, including AEs, vital signs, body weight, and safety laboratory. Pooled data across the two treatment periods were also summarized not adjusting for sequence and period to compare the change in body weight, BNP, hemoglobin, and BP at week 12 between atrasentan and placebo.

The analyses were performed using SAS version 9.4 (SAS Institute, Inc, Cary, NC). A two-sided *P* value < 0.05 was considered significant.

## Results

### Patient Disposition and Characteristics

**Figure 2:**
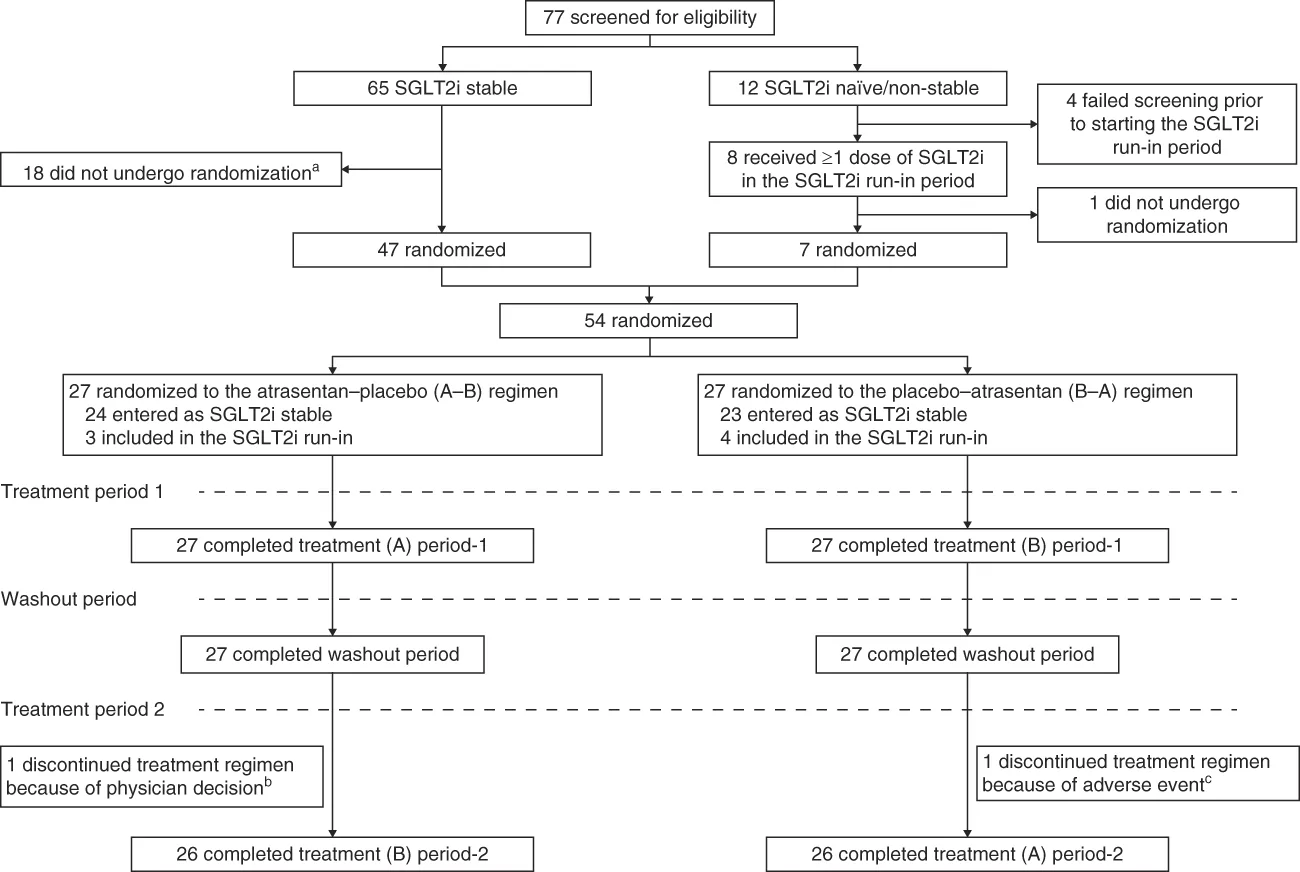
**Patient disposition.** ^a^Participants did not undergo randomization because of screening failure. ^b^Participant discontinued treatment due to inability to continue with study visits and discontinued from the study. ^c^Participant discontinued treatment but completed the study.

From November 2023 to October 2024, 77 individuals were screened and assessed for eligibility, of whom 54 were randomized (Figure 2). All randomized participants received at least one dose of study medication, resulting in 54 participants in the intention-to-treat and safety analysis. All 54 participants completed treatment period 1 and the washout period. Fifty-two participants completed treatment period 2. One participant (1/27, 4%) in the atrasentan-to-placebo sequence discontinued the study treatment during period 2 due to inability to continue with study visits and also discontinued early from the study. One (1/27, 4%) participant in the placebo-to-atrasentan sequence discontinued treatment in period 2 due to an unrelated serious AE but completed the study.

Baseline characteristics were generally well balanced across the randomized treatment sequences (Table 1), apart from some numerical differences in age and BP. The mean (SD) age of participants was 48 (12) years; 23 (43%) participants were female, and 35 (65%) were White and 15 (28%) Asian. The mean (SD) eGFR was 63 (22) ml/min per 1.73 m^2^, and the median (25th–75th percentile) UPCR was 1.0 g/g (0.7–1.4). Most participants (*n*=47; 87%) were receiving stable doses of SGLT2 inhibitors before study entry, while *n*=7 (13%) entered the SGLT2 inhibitor run-in period. Adherence to study medication was excellent, with 95% and 97% of study tablets taken during the first and second treatment period, respectively.

**Table 1:** Baseline demographics and disease characteristics of patients with IgA nephropathy^a^^,^^b^^,^^c^

Characteristic	Treatment Period 1			Treatment Period 2		
	Atrasentan (*N*=27)	Placebo (*N*=27)	Total (*N*=54)	Placebo (*N*=27)	Atrasentan (*N*=27)	Total (*N*=54)
Age, yr, mean (SD)	52 (11)	43 (11)	48 (12)	—	—	—
Age group, <65 yr, *n* (%)	23 (85)	26 (96)	49 (91)	—	—	—
Sex, women, *n* (%)	12 (44)	11 (41)	23 (43)	—	—	—
**Race, ** ***n*** ** (%)**						
Asian	10 (37)	5 (19)	15 (28)	—	—	—
Multiple	1 (4)	2 (7)	3 (6)	—	—	—
Not reported	1 (4)	0	1 (2)	—	—	—
White	15 (56)	20 (74)	35 (65)	—	—	—
**Geographic region, ** ***n*** ** (%)**						
Asia	8 (30)	7 (26)	15 (28)	—	—	—
Europe	5 (19)	5 (19)	10 (19)	—	—	—
Latin America	7 (26)	12 (44)	19 (35)	—	—	—
North America	2 (7)	2 (7)	4 (7)	—	—	—
Oceania	5 (19)	1 (4)	6 (11)	—	—	—
Duration of disease^d^, yr, median (Q1–Q3)	4.9 (0.8–10.0)	4.9 (2.2–7.9)	4.9 (2.0–10.0)	—	—	—
BMI^e^, kg/m^2^, mean (SD)	30.0 (6.4)	29.2 (5.7)	29.6 (6.0)	30.1 (6.5)	28.9 (5.1)	29.5 (5.8)
**BP** ^f^ **, mm Hg, mean (SD)**						
Systolic	126 (16)	124 (15)	125 (15)	126 (14)	122 (15)	124 (14)
Diastolic	77 (9)	76 (13)	76 (11)	78 (11)	74 (10)	76 (10)
24-h total urine protein, g/d, median (Q1–Q3)	1.5 (0.9–2.4)	1.8 (1.1–3.0)	1.6 (1.0–2.5)	1.2 (0.9–2.4)	1.8 (0.8–3.2)	1.3 (0.9–2.6)
**24-** **hour** ** UPCR, g/g**						
Median (Q1–Q3)	0.9 (0.7–1.4)	1.1 (0.7–1.5)	1.0 (0.7–1.4)	1.0 (0.7–1.5)	1.0 (0.5–1.6)	1.0 (0.6–1.5)
Distribution, *n* (%)						
*<1.5*	23 (85)	20 (74)	43 (79.6)	21 (78)	18 (67)	39 (72)
*≥1.5*	4 (15)	7 (26)	11 (20)	6 (22)	9 (33)	15 (28)
24-h UACR, g/g, median (Q1–Q3)	0.6 (0.5–1.0)	0.6 (0.5–1.1)	0.6 (0.5–1.0)	0.6 (0.5–0.9)	0.7 (0.3–1.1)	0.7 (0.4–1.0)
**eGFR, ml/min per 1.73 m** ^**2**^						
Mean (SD)	63 (20)	64 (24)	63 (22)	61 (21)	62 (25)	61 (23)
Distribution, *n* (%)						
*≤45*	6 (22)	8 (30)	14 (26)	7 (26)	12 (44)	19 (35)
*>45 to ≤60*	9 (33)	6 (22)	15 (28)	9 (33)	3 (11)	12 (22)
*>60*	12 (44)	13 (48)	25 (46)	11 (41)	12 (44)	23 (43)
BNP, ng/l, mean (SD)	20 (18)	17 (16)	19 (17)	16 (12)	24 (38)	20 (28)
**Use of RASi at baseline, ** ***n*** ** (%)**						
ACEi only	9 (33)	10 (37)	19 (35)	—	—	—
ARB only	18 (67)	17 (63)	35 (65)	—	—	—
**Baseline medications of special interest, ** ***n*** ** (%)**						
RASi	27 (100)	27 (100)	54 (100)	27 (100)	27 (100)	54 (100)
SGLT2 inhibitor	27 (100)	27 (100)	54 (100)	27 (100)	27 (100)	54 (100)
BP-lowering agents^g^	16 (59)	8 (30)	24 (44)	16 (59)	9 (33)	25 (46)
Diuretics	6 (22)	11 (41)	17 (31)	6 (22)	12 (44)	18 (33)
Immunosuppressants^h^	1 (4)	0	1 (2)	1 (4)	0	1 (2)

ACEi, angiotensin-converting enzyme inhibitor; ARB, angiotensin receptor blocker; BMI, body mass index; BNP, B-type natriuretic peptide; CI, confidence interval; Q, quartile; RASi, renin-angiotensin system inhibitors; SGLT2, sodium-glucose cotransporter 2; UACR, urinary albumin-to-creatinine ratio; UPCR, urinary protein-to-creatinine ratio.

a Eight patients had missing data for the primary end point.

b To facilitate within sequence comparisons, the placebo column is presented first in treatment period 2.

c Age, sex, geographic region, duration of disease, and use of renin-angiotensin system inhibitors at baseline were assessed only at baseline 1 at the start of treatment period 1.

d Duration of disease is defined as the time from the most recent biopsy (first dose date in treatment period 1 – most recent biopsy date)/365.25.

e Body mass index was derived from height at screening and weight at each visit, based on the formula: body mass index=weight (kg)/height (m^2^).

f Baseline value is based on the BP value collected at the last assessment on or before study day 1.

g One patient was on a combination pill for antihypertensive therapy that included a diuretic.

h One participant was on an immunosuppressant (for inflammatory bowel disease) that is not considered to be usual therapy for IgA nephropathy.

### Primary Efficacy End Point: 24-Hour UPCR

**Figure 3:**
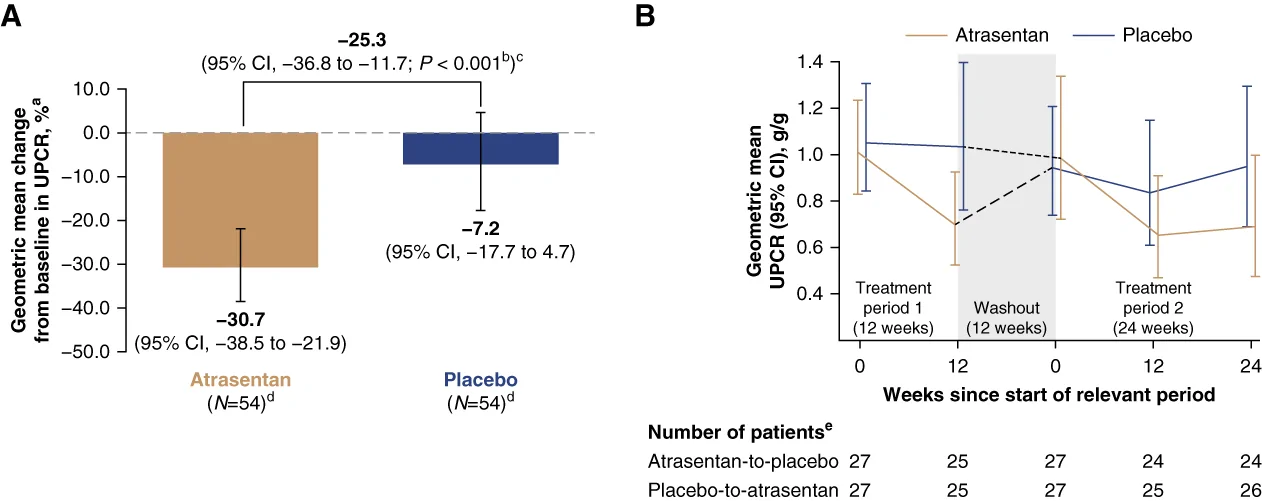
**Twenty-four–hour UPCR (primary outcome).** (A) Change in 24-hour UPCR from baseline to week 12. (B) 24-hour UPCR by visit. ^a^UPCR in a logarithmic scale change from baseline (within each treatment period) was analyzed based on mixed model with sequence, period, and treatment as fixed-effect factors, and baseline natural log UPCR and the randomization stratification factor of SGLT2 inhibitor status (stable versus run-in) as covariates, with a random effect of participants nested within sequences. Rubin's rules were applied to the estimates from multiple imputations. ^b^Indicates two-sided nominal *P* value < 0.05. ^c^The difference in atrasentan and placebo is derived from a within-participant comparison. ^d^Intercurrent event handling strategy: hypothetical strategy applied to “Initiation of an investigational or approved product for the treatment of IgA nephropathy (other than RASi and SGLT2 inhibitor)”; treatment policy applied to the other intercurrent events (study drug discontinuation for any reason, initiation of maintenance dialysis or kidney transplantation, dose modification or discontinuation of background therapy [RASi/SGLT2i], initiation of prohibited or restricted concomitant medication). Intention-to-treat analysis set includes the number of participants with nonmissing observation. ^e^At each postbaseline visit, only patients with a value at both baseline and that timepoint were included. CI, confidence interval.

After 12 weeks of treatment with atrasentan and placebo, geometric mean percentage change from baseline in UPCR was −30.7% (95% confidence interval [CI], −38.5 to −21.9) and −7.2% (95% CI, −17.7 to 4.7), respectively (Figure 3A). Accordingly, the difference between atrasentan and placebo in the change from baseline in UPCR was −25.3% (95% CI, −36.8 to −11.7; *P* < 0.001). Twelve weeks after the discontinuation of atrasentan or placebo during treatment period 1, UPCR had returned to baseline levels (Figure 3B). In the atrasentan-to-placebo sequence, the change in UPCR from baseline to week 12 was −30.5% (95% CI, −41.3 to −17.7) during atrasentan treatment and −11.7% (95% CI, −25.6 to 4.9) during placebo treatment. The corresponding UPCR change in the placebo-to-atrasentan sequence was −31.1% (95% CI, −41.6 to −18.6) during atrasentan treatment and −2.3% (95% CI, −17.5 to 15.6) during placebo treatment. To assess the robustness of the primary outcome results and the impact of intercurrent events, prespecified sensitivity analyses were performed, which showed similar results to the main analysis (Supplemental Tables 2–4).

To determine whether treatment sequence influenced the degree of proteinuria reduction, the geometric mean ratio of 24-hour UPCR at week 12 in each treatment period versus baseline was assessed. There was no clear indication of a carryover effect; for the geometric adjusted mean ratios of 24-hour UPCR at week 12 versus baseline, the within-individual ratios of atrasentan versus placebo were 0.8 (95% CI, 0.6 to 1.0) and 0.7 (95% CI, 0.6 to 0.9) for patients receiving the atrasentan-to-placebo treatment sequence and placebo-to-atrasentan treatment sequence, respectively (Supplemental Table 5).

### Secondary Efficacy End Point

At the start of treatment period 2, the baseline median UPCR was 1.0 g/g (25th–75th percentile 0.7–1.5) for those in the atrasentan-to-placebo sequence and 1.0 g/g (25th–75th percentile 0.5–1.6) for those in the placebo-to-atrasentan sequence. The geometric mean percentage change from baseline in UPCR at week 24 during treatment period 2 was −27.0% (95% CI, −41.0 to −9.8) for those receiving atrasentan and −0.9% (95% CI, −20.6 to 23.7) for those receiving placebo, corresponding to a treatment difference of −26.4% (95% CI, −45.8 to −0.0).

### Exploratory End Points

**Figure 4:**
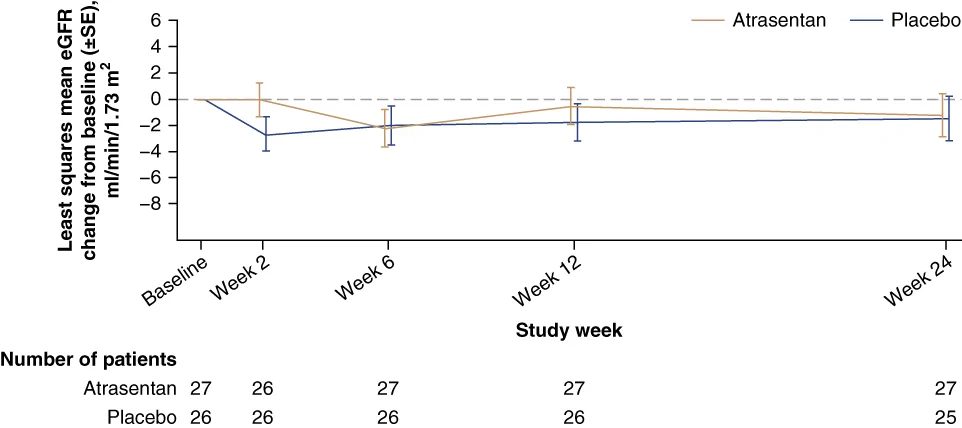
Change from baseline in eGFR over 24 weeks (treatment period 2; exploratory outcome).

At the start of treatment period 2, the mean eGFR in the atrasentan-to-placebo sequence was 61 (SD 21) ml/min per 1.73 m^2^; in the placebo-to-atrasentan sequence, it was 62 (SD 25) ml/min per 1.73 m^2^. The least squares mean change from baseline at week 24 was −1.2 ml/min per 1.73 m^2^ (95% CI, −4.6 to 2.1) for the placebo-to-atrasentan sequence and −1.5 ml/min per 1.73 m^2^ (95% CI, −4.9 to 2.0) for the atrasentan-to-placebo sequence, corresponding to a between-group difference of 0.3 ml/min per 1.73 m^2^ (95% CI, −4.5 to 5.0). eGFR remained stable during 12 and 24 weeks of treatment with atrasentan and placebo throughout the study, regardless of treatment period (Figure 4 and Supplemental Figure 1).

### Safety

In treatment period 1, TEAEs were reported in 19 (70%) participants during atrasentan treatment and 17 (63%) during placebo treatment (Table 2 and Supplemental Table 6). Most of these AEs were mild, with equivalent numbers of participants having AEs of moderate severity during both atrasentan (7, 26%) and placebo (7, 26%) treatment. During treatment period 1, one participant had an AE of AKI while on atrasentan, reported as moderate in severity and deemed related to study drug and auxiliary medications. There were no severe or serious AEs during treatment period 1. During period 2, TEAEs were reported in 16 participants (59%) during atrasentan and 17 (63%) during placebo treatment. One participant had an AE of dizziness reported as moderate in severity and related to study drug while on atrasentan treatment. During treatment period 2, there was one serious AE of a severe cytomegalovirus infection in an atrasentan-treated participant.

**Table 2:** Treatment-emergent adverse events

TEAE, *n* (%)	Treatment Period 1		Treatment Period 2	
	Atrasentan (*N*=27)	Placebo (*N*=27)	Placebo (*N*=27)	Atrasentan (*N*=27)
**Any TEAE**	19 (70)	17 (63)	17 (63)	16 (59)
Treatment-related	6 (22)	4 (15)	3 (11)	2 (7)
**Any ** **moderate/severe** ** TEAEs**	7 (26)	7 (26)	7 (26)	7 (26)
Treatment-related	1 (4)	0	0	1 (4)
**Any severe TEAEs** ^a^	0	0	0	1 (4)
Treatment-related	0	0	0	0
**Any serious TEAEs** ^a^	0	0	0	1 (4)
Treatment-related	0	0	0	0
**TEAEs leading to treatment discontinuation** ^a^	0	0	0	1 (4)
Treatment-related	0	0	0	0
TEAE related to auxiliary medications^b^	1 (4)	0	3 (11)	2 (7)
TEAEs with a fatal outcome	0	0	0	0

A participant with multiple occurrences for an adverse event was counted only once. Treatment-emergent adverse events are defined as adverse events that first occurred or worsened on or after the first dose of treatment in each randomized treatment period and within 30 days after the last dose of treatment in each period. MedDRA version 28.1 was used for the adverse event reporting. TEAE, treatment-emergent adverse events.

a One patient experienced a serious treatment-emergent adverse event of severe cytomegalovirus infection deemed unrelated to treatment that resulted in study drug discontinuation.

b Auxiliary medications were defined as authorized angiotensin-converting enzyme inhibitor/angiotensin receptor blocker and sodium-glucose cotransporter 2 inhibitor.

TEAESIs were all mild or moderate in severity and none were severe. There were no serious TEAESIs during the study. In the first treatment period, TEAESIs were reported in six (22%) atrasentan-treated and two (7%) placebo-treated participants (Table 3). The corresponding numbers in the second treatment period were four (15%) and six (22%) participants, respectively. In treatment period 1, treatment-emergent fluid retention events occurred in five participants (19%) on atrasentan and one (4%) on placebo. In treatment period 2, treatment-emergent fluid retention events occurred in three participants (11%) on atrasentan and three (11%) on placebo. No deaths or heart failure events occurred during the study.

**Table 3:** Treatment-emergent adverse events of special interest by maximum severity

TEAESI Category (Preferred Term), *n* (%)	Treatment Period 1		Treatment Period 2	
	Atrasentan (*N*=27)	Placebo (*N*=27)	Placebo (*N*=27)	Atrasentan (*N*=27)
**TEAESIs** ^a^	6 (22)	2 (7)	6 (22)	4 (15)
Treatment-related	5 (19)	1 (4)	3 (11)	1 (4)
Serious TEAESIs	0	0	0	0
**Fluid retention** ^b^	5 (19)	1 (4)	3 (11)	3 (11)
Peripheral edema	1 (4)	1 (4)	1 (4)	1 (4)
*Mild*	1 (4)	1 (4)	1 (4)	1 (4)
*Moderate*	0	0	0	0
*Severe*	0	0	0	0
Fluid retention	3 (11)	0	3 (11)	1 (4)
*Mild*	3 (11)	0	3 (11)	0
*Moderate*	0	0	0	1 (4)
*Severe*	0	0	0	0
Peripheral swelling	0	0	0	1 (4)
*Mild*	0	0	0	1 (4)
*Moderate*	0	0	0	0
*Severe*	0	0	0	0
Edema	1 (4)	0	0	0
*Mild*	1 (4)	0	0	0
*Moderate*	0	0	0	0
*Severe*	0	0	0	0
Anemia	1 (4)	0	0	0
*Mild*	0	0	0	0
*Moderate*	1 (4)	0	0	0
*Severe*	0	0	0	0
**Vasodilation/hypotension**	1 (4)	1 (4)	3 (11)	2 (7)
Hypotension	1 (4)	1 (4)	3 (11)	1 (4)
*Mild*	1 (4)	1 (4)	2 (7)	0
*Moderate*	0	0	1 (4)	1 (4)
*Severe*	0	0	0	0
Orthostatic hypotension	0	0	0	1 (4)
*Mild*	0	0	0	1 (4)
*Moderate*	0	0	0	0
*Severe*	0	0	0	0

TEAESI, treatment-emergent adverse events of special interest.

a Treatment-emergent adverse events are defined as adverse events that first occurred or worsened on or after the first dose of treatment in each randomized treatment period and within 30 days after the last dose of treatment in each period. Adverse event of special interests were identified using Office of New Drugs Custom Medical Queries (OCMQ) peripheral edema-narrow and broad (fluid retention), OCMQ anemia-narrow (anemia), OCMQ hypotension-narrow (vasodilation/hypotension), and OCMQ heart failure-narrow (cardiac failure).

b While two participants with fluid retention received diuretics in treatment period 1, only one of these participants was receiving atrasentan (atrasentan-placebo sequence) and the other was receiving placebo (placebo-atrasentan sequence). One participant receiving atrasentan in treatment period 2 received a loop diuretic (placebo-atrasentan sequence). For all participants receiving diuretics, the fluid retention treatment-emergent adverse events of special interest resolved.

**Figure 5:**
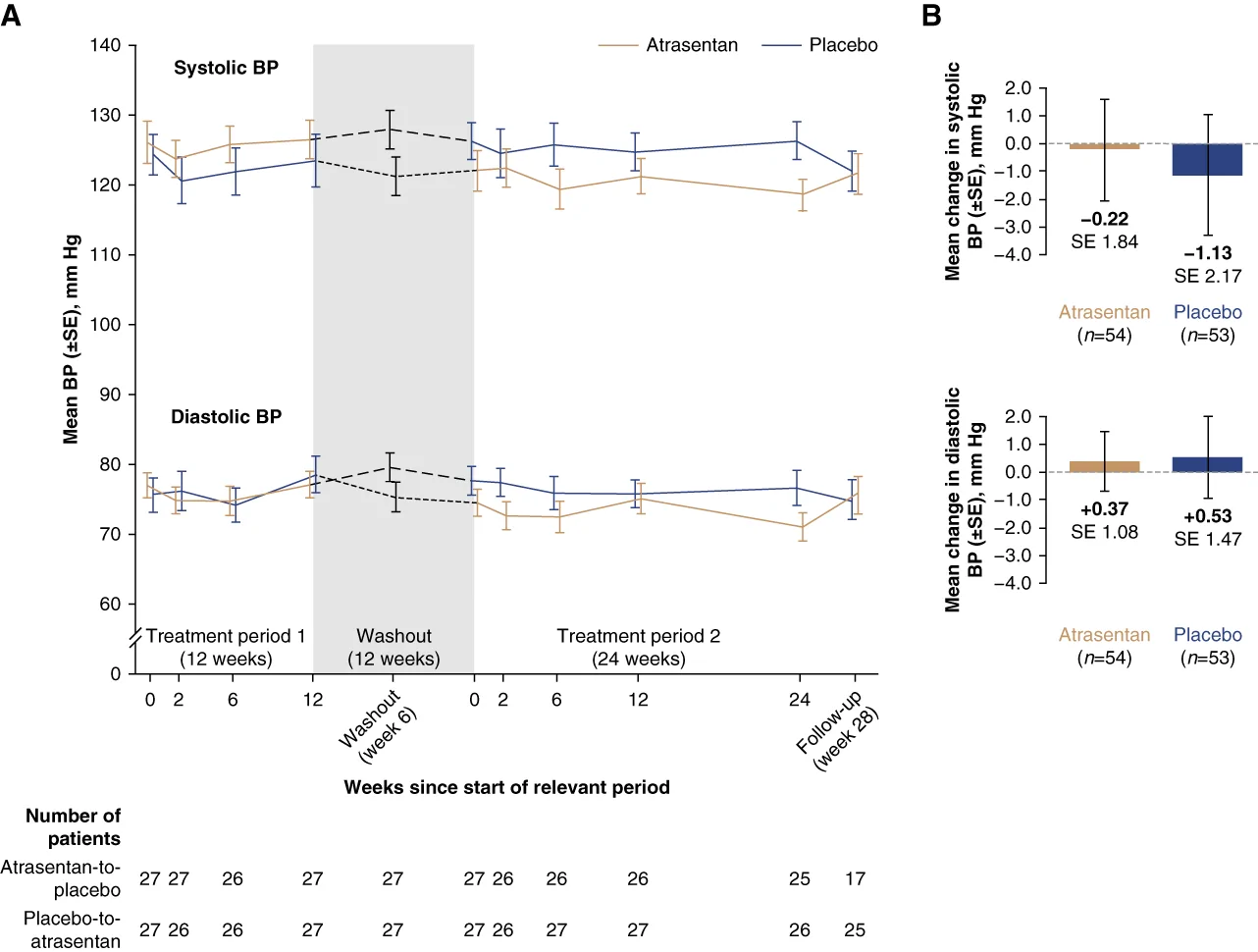
**Systolic and diastolic BP over time.** (A) Longitudinal data over the course of the study (prespecified data collection). (B) Change from baseline to week 12: pooled data by allocated treatment (including both treatment periods; *post hoc* analysis).

BP values were relatively stable throughout the study (Figure 5A). In treatment period 1, mean systolic BP at baseline was 126 mm Hg (SD 16) in the atrasentan-to-placebo sequence, and by week 12, the mean change in systolic BP was 0.4 mm Hg (SD 13). In the placebo-to-atrasentan sequence during period 1, mean systolic BP at baseline was 124 mm Hg (SD 15), and by week 12, the mean change in systolic BP was −0.9 mm Hg (SD 20). In treatment period 2, in the atrasentan-to-placebo sequence, by week 24, the mean change in systolic BP was −0.2 mm Hg (SD 12). In treatment period 2, in the placebo-to-atrasentan sequence, by week 24, the mean change in systolic BP was −3.7 mm Hg (SD 12). Similar patterns of minimal changes from baseline in diastolic BP were observed during treatment period 1 and period 2 (Figure 5A).

**Figure 6:**
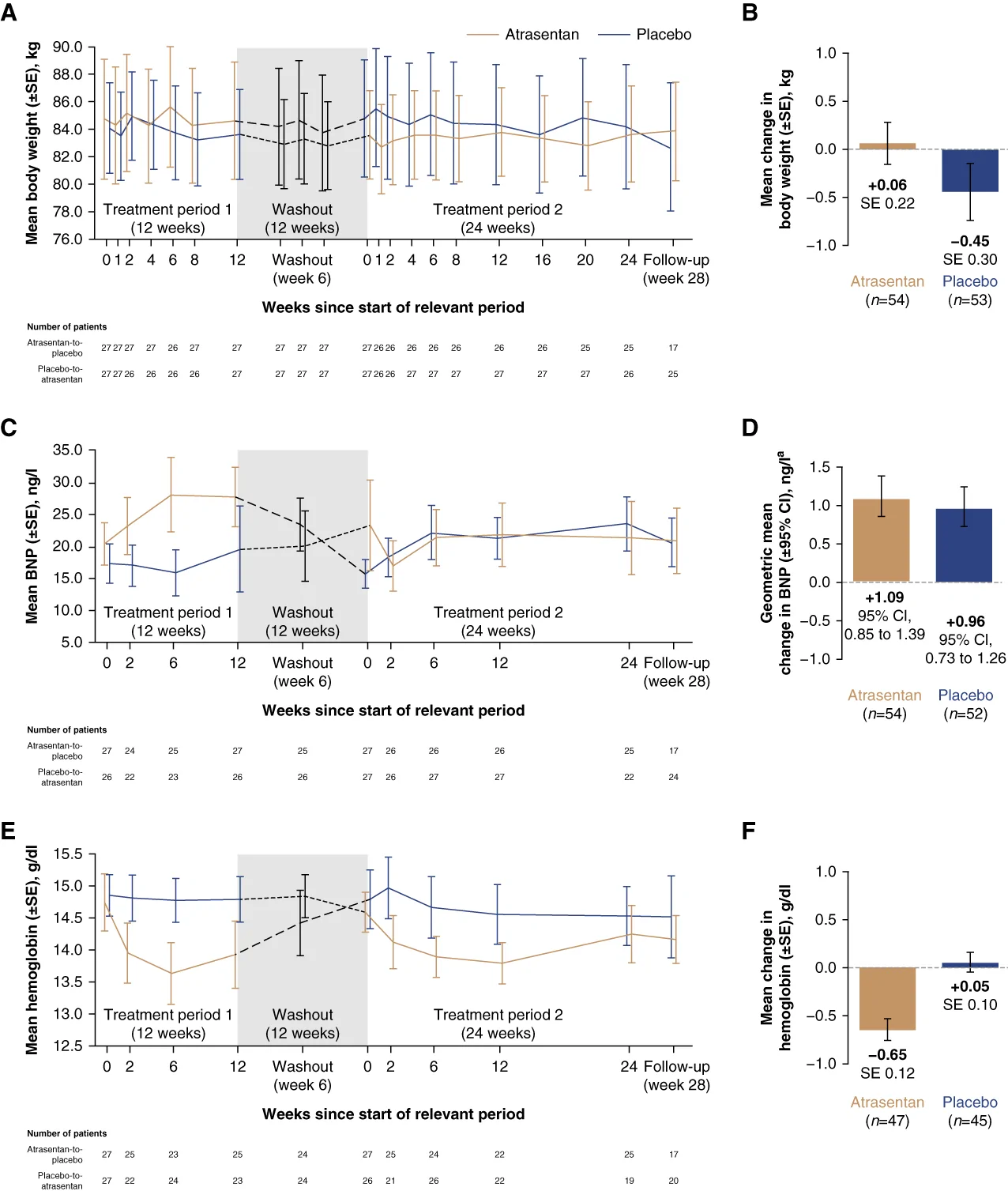
**Body weight, BNP, and hemoglobin levels over time.** (A) Body weight: longitudinal data over the course of the study (prespecified data collection). (B) Body weight: change from baseline to week 12: pooled data by allocated treatment (including both treatment periods; *post hoc* analysis). (C) BNP: longitudinal data over the course of the study (prespecified data collection). (D) BNP: change from baseline to week 12: pooled data by allocated treatment (including both treatment periods; *post hoc* analysis). (E) Hemoglobin: longitudinal data over the course of the study (prespecified data collection). (F) Hemoglobin: change from baseline to week 12: pooled data by allocated treatment (including both treatment periods; *post hoc* analysis). ^a^Geometric mean is presented as the mean BNP values had a skewed distribution. BNP, B-type natriuretic peptide.

Body weight, BNP, and hemoglobin values were assessed as proxies for fluid retention (Figure 6). Body weight did not differ at baseline in either treatment sequence and remained stable throughout both treatment periods. In treatment period 1, at week 12, the mean change from baseline in body weight was −0.1 kg (SD 1.5) with atrasentan treatment and −0.5 kg (SD 2.5) with placebo. In treatment period 2, at week 24, the mean change from baseline in body weight was −0.1 kg (SD 3.4) with placebo treatment and 0.1 kg (SD 3.1) with atrasentan (Figure 6A). BNP levels were similar between both sequences at baseline and well below the upper limit of normal (100 ng/L) (Figure 6C). The mean hemoglobin slightly decreased during atrasentan treatment, <1 g/dl, and remained stable during the placebo treatment (Figure 6E).

*Post hoc* analysis pooling data from the two treatment periods with atrasentan (*N=*54) or placebo (*N=*54) to assess the overall change from baseline in body weight, BNP, and hemoglobin showed no meaningful difference between atrasentan and placebo treatments (Figure 6).

## Discussion

In this randomized, double-blind, placebo-controlled crossover study in participants with IgA nephropathy receiving SGLT2 and maximal tolerated RAS inhibition, 12 weeks of treatment with the selective ETA receptor antagonist atrasentan resulted in a 25% larger reduction in UPCR than placebo. These results were maintained at 24 weeks in treatment period 2, and eGFR remained stable throughout both treatment periods, regardless of treatment assignment. Although there was a modest imbalance in fluid retention TEAESIs in treatment period 1, which was accompanied by a slight decrease in hemoglobin during atrasentan treatment, none of the TEAESIs were severe and none led to hospitalization. The robust and clinically meaningful reduction in UPCR and acceptable safety profile provide initial support of atrasentan as a potential adjunct treatment in the care of patients with IgA nephropathy and persistent proteinuria despite standard of care with SGLT2 and RAS inhibition. The SGLT2 inhibitor stratum of the ALIGN trial will provide more insights into the longer-term efficacy and safety of atrasentan when added to SGLT2 inhibitors.^13^

These findings build on the growing body of evidence supporting the favorable benefit–risk profile of combining ERAs with SGLT2 inhibitors. A small cohort study in patients with type 2 diabetes and CKD suggested that simultaneous initiation of atrasentan with SGLT2 inhibition may further reduce albuminuria compared with atrasentan alone without causing fluid retention.^10^ The Zibotentan and Dapagliflozin for the Treatment of CKD trial showed that low-dose zibotentan in combination with dapagliflozin resulted in a significant 30% reduction in albuminuria with minimal signs of fluid retention.^14^ In an exploratory analysis of the ALIGN trial, in 29 patients with IgA nephropathy who were using RAS and SGTL2 inhibitors, at interim analysis, addition of atrasentan reduced proteinuria to a similar degree compared with 270 participants not using SGLT2 inhibition^7^; this may suggest that the treatments reduce proteinuria through different yet complementary mechanisms.^5^ The current findings provide adequately powered, prospective evidence of the additive contributions of atrasentan in conjunction with RAS and SGLT2 inhibitors. The observed 25% reduction in proteinuria with atrasentan added to maximal background therapy is clinically meaningful. Based on a meta-analysis of clinical trials, a 25% reduction in albuminuria is expected to provide more than 97.5% confidence that an intervention will reduce the risk of kidney failure.^12^

The ASSIST trial population had less severe CKD in comparison with the ALIGN trial as reflected by a lower median UPCR of 1.0 g/g versus 1.4 g/g.^7^ In addition, 26% of ASSIST participants had an eGFR <45 ml/min per 1.73 m^2^ compared with 40% in ALIGN.^7^ The clinically significant reduction in UPCR in ASSIST, despite a relatively milder CKD phenotype, would support the initiation of atrasentan at proteinuria levels below the traditional target threshold of 1.0 g/d to delay long-term kidney disease progression.

Treatment period 2 of the ASSIST trial was extended beyond 12 weeks to study the time course of the effect of atrasentan on proteinuria in a well-designed, placebo-controlled, double-blind trial. In the ASSIST trial, the effects of atrasentan were fully present at 12 weeks with no further UPCR reduction at 24 weeks' treatment. These results are consistent with the ALIGN trial, which demonstrated that most of the reduction in UPCR occurred within the first 6 weeks after initiating atrasentan.^7^ The rapid onset of this effect suggests involvement of hemodynamic factors.

Additional mechanistic studies to evaluate the effects of atrasentan on kidney hemodynamics in more detail may demonstrate atrasentan effects beyond the commonly recognized hemodynamic impact. The results of the SGLT2 inhibitor cohort analysis in the ALIGN trial will provide more evidence on whether combination therapy of atrasentan and SGLT2 inhibition serves to reduce proteinuria and kidney function loss and minimize potential long-term volume-related side effects.

There were no clinically significant changes in BP, body weight, BNP, or hemoglobin with either atrasentan or placebo treatment throughout the study. Regarding fluid retention–related AEs with atrasentan and concomitant SGLT2i, the small sample size in this study limits definitive conclusions, yet the findings are reassuring because the events were well managed without requiring treatment discontinuation.

Strengths of this study include a high retention rate, with no participants withdrawing from the study during treatment period 1 and only one participant withdrawing during treatment period 2. Adherence to study drug was excellent: 95% and 97% during treatment periods 1 and 2, respectively. The crossover study design adds to the robustness of our results by eliminating inter-participant variability and maximizing statistical power. There are also limitations to this study, however, including the relatively small number of participants, short study duration, and use of a surrogate end point, and therefore, the exploratory eGFR results should be interpreted with caution. Proteinuria is now a well-vetted, reasonably likely surrogate end point for IgA nephropathy, with several meta-analyses demonstrating high correlation between proteinuria reduction and improved clinical kidney end points.^12,15,16^

In conclusion, this randomized, placebo-controlled crossover study shows the added benefit in reducing proteinuria and safety of the selective ETA receptor antagonist atrasentan to background therapy of IgA nephropathy with both SGLT2 and RAS inhibition. Based on these results, atrasentan may be considered an adjunctive therapy in patients with IgA nephropathy and proteinuria >0.5 g/d.

## Supplementary Material

## Data Availability

Data belong to a third party, and authors are not authorized to share the data. Identity of Third Party: Novartis. Reason for Restriction: Novartis is committed to sharing with qualified external researchers access to patient-level data and supporting clinical documents from eligible studies. These requests are reviewed and approved by an independent review panel on the basis of scientific merit. All data provided are anonymized to respect the privacy of patients who have participated in the trial in line with applicable laws and regulations. This trial data availability is according to the criteria and process described on www.clinicalstudydatarequest.com.

## Declarative Statements

This study includes clinical experimentation and received Institutional Review Board or Ethics Committee approval. All patients provided written informed consent. This study includes clinical experimentation and complies with the Declaration of Helsinki.

## Supplemental Material

List of ASSIST steering committee members.

List of ASSIST principal investigators.

Supplemental Methods.

Supplemental Table 1. Methodology for supplementary analyses of the primary end point.

Supplemental Table 2. Prespecified supplementary analysis 1 of the primary end point: change from baseline to week 12 in 24-hour UPCR.

Supplemental Table 3. Prespecified supplementary analysis 2 of the primary end point: change from baseline to week 12 in 24-hour UPCR.

Supplemental Table 4. Prespecified supplementary analysis 3 of the primary end point: change from baseline to week 12 in 24-hour UPCR.

Supplemental Table 5. Geometric mean ratio of 24-hour UPCR at week 12 versus baseline within each treatment period.

Supplemental Table 6. Most common treatment-emergent AEs (TEAEs; >5% of patients).

Supplemental Figure 1. eGFR by visit (safety analysis set).

Statistical Analysis Plan.

## References

[1] Xie J Kiryluk K Wang W, . Predicting progression of IgA nephropathy: new clinical progression risk score. PLoS One. 2012;7(6):e38904. doi:10.1371/journal.pone.0038904 22719981 PMC3375310

[2] Pitcher D Braddon F Hendry B, . Long-term outcomes in IgA nephropathy. Clin J Am Soc Nephrol. 2023;18(6):727–738. doi:10.2215/CJN.0000000000000135 37055195 PMC10278810

[3] Rovin BH Barratt J Cook HT, . Kidney Disease Improving Global Outcomes KDIGO IgAN and IgAV Work Group. KDIGO 2025 clinical practice guideline for the management of immunoglobulin A nephropathy (IgAN) and immunoglobulin A vasculitis (IgAV). Kidney Int. 2025;108(4S):S1–S71. doi:10.1016/j.kint.2025.04.004 40975564

[4] Smeijer JD Kohan DE Webb DJ Dhaun N Heerspink HJL. Endothelin receptor antagonists for the treatment of diabetic and nondiabetic chronic kidney disease. Curr Opin Nephrol Hypertens. 2021;30(4):456–465. doi:10.1097/mnh.0000000000000716 33990507

[5] Smeijer JD Kohan DE Dhaun N Noronha IL Liew A Heerspink HJL. Endothelin receptor antagonists in chronic kidney disease. Nat Rev Nephrol. 2025;21(3):175–188. doi:10.1038/s41581-024-00908-z 39643698

[6] Kuo JJ Wu J Gunawan MG, . Effects of atrasentan on kidney gene transcription, mesangial cell proliferation, and proteinuria in immunoglobulin A nephropathy. Kidney360. 2025;5(6):802–811. doi:10.34067/kid.0000001000 PMC13229432 41442197

[7] Heerspink HJL Jardine M Kohan DE, . Atrasentan in patients with IgA nephropathy. N Engl J Med. 2025;392(6):544–554. doi:10.1056/NEJMoa2409415 39460694

[8] Novartis Pharmaceuticals. VANRAFIA™ (Atrasentan) Tablets, for Oral Use: Highlights of Prescribing Information. 2025. Accessed January 20, 2026 https://www.novartis.com/us-en/sites/novartis_us/files/vanrafia.pdf.

[9] Lambers Heerspink HJ de Zeeuw D Wie L Leslie B List J. Dapagliflozin a glucose-regulating drug with diuretic properties in subjects with type 2 diabetes. Diabetes Obes Metab. 2013;15(9):853–862. doi:10.1111/dom.12127 23668478 PMC3906841

[10] Heerspink HJL Kohan DE de Zeeuw D. New insights from SONAR indicate adding sodium glucose co-transporter 2 inhibitors to an endothelin receptor antagonist mitigates fluid retention and enhances albuminuria reduction. Kidney Int. 2021;99(2):346–349. doi:10.1016/j.kint.2020.09.026 33144213

[11] Mottl AK Mastroianni-Kirsztajn G Noronha IL, . Baseline characteristics of ASSIST: phase 2 crossover trial of atrasentan in adults with IgAN on SGLT2 inhibitors: FR-PO0831. J Am Soc Nephrol. 2025;36(10S). doi:10.1681/ASN.20251m9dyn78

[12] Heerspink HJL Collier WH Chaudhari J, . A meta-analysis of albuminuria as a surrogate endpoint for kidney failure. Nat Med. 2026;32(1):281–287. doi:10.1038/s41591-025-04057-z 41198855

[13] Heerspink HJL Jardine M Kohan DE, . Study design and baseline characteristics of ALIGN, a randomized controlled study of atrasentan in patients with IgA nephropathy. Kidney Int Rep. 2025;10(1):217–226. doi:10.1016/j.ekir.2024.10.004 39810771 PMC11725817

[14] Heerspink HJL Kiyosue A Wheeler DC, . Zibotentan in combination with dapagliflozin compared with dapagliflozin in patients with chronic kidney disease (ZENITH-CKD): a multicentre, randomised, active-controlled, phase 2b, clinical trial. Lancet. 2023;402(10416):2004–2017. doi:10.1016/s0140-6736(23)02230-4 37931629

[15] Thompson A Carroll K A Inker L, . Proteinuria reduction as a surrogate end point in trials of IgA nephropathy. Clin J Am Soc Nephrol. 2019;14(3):469–481. doi:10.2215/CJN.08600718 30635299 PMC6419287

[16] Inker LA Heerspink HJL Tighiouart H, . Association of treatment effects on early change in urine protein and treatment effects on GFR slope in IgA nephropathy: an individual participant meta-analysis. Am J Kidney Dis. 2021;78(3):340–349.e1. doi:10.1053/j.ajkd.2021.03.007 33775708 PMC8384669

