# Supplementary material for: Efficacy and Safety of Atrasentan in Patients with IgA Nephropathy Receiving Sodium-Glucose Cotransporter 2 Inhibitors: Placebo-Controlled, Crossover Trial
Source: J Am Soc Nephrol. 2026 Mar 29;37(9):2004–15. doi: 10.1681/ASN.0000001076 (PMC13567876; doi:10.1681/ASN.0000001076)
Supplement: Supplementary file 1 [file jasn-37-2004-s001.pdf]

ASN American Society of Nephrology
JASN Journal of the American Society of Nephrology
KIDNEY360 Accessing Our World From Every Angle
CJASN Clinical Journal of the American Society of Nephrology

ASN Journal Disclosure Form

As per ASN journal policy, I have disclosed any financial relationships or commitments I have held in the past 36 months as included below. I have listed my Current Employer below to indicate there is a relationship requiring disclosure. If no relationship exists, my Current Employer is not listed.

J. Barratt reports the following:
Employer: UNIVERSITY OF LEICESTER; Consultancy: Alexion, Arrowhead, Astellas, Alebund, Alnylam, Alpine, Argenx, BioCryst, Calliditas, Chinook, Dimerix, HiBio, Kira, Novartis, Omeros, Otsuka, Travere Therapeutics, Q32 Bio, Roche, Sanofi, Takeda, Vera Therapeutics, CSL Vifor, Visterra; Research Funding: Alexion, Novartis; GlaxoSmithKline; Calliditas, Visterra, Chinook, Omeros, Galapagos, argenx, Travere Therapeutics; and Advisory or Leadership Role: Editorial Board of Kidney International, CJASN, Glomerular Diseases & Clinical Science; Convener International IgA Nephropathy Network.

I understand that the information above will be published within the journal article, if accepted, and that failure to comply and/or to accurately and completely report the potential financial conflicts of interest could lead to the following: 1) Prior to publication, article rejection, or 2) Post-publication, sanctions ranging from, but not limited to, issuing a correction, reporting the inaccurate information to the authors' institution, banning authors from submitting work to ASN journals for varying lengths of time, and/or retraction of the published work.

Name: Jonathan Barratt
Manuscript ID: JASN-2026-000190
Manuscript Title: Atrasentan reduces proteinuria in patients with IgA nephropathy receiving sodium–glucose co-transporter-2 inhibitors: A randomized, double-blind, placebo-controlled crossover trial
Date of Completion: February 11, 2026
Disclosure Updated Date: February 11, 2026

ASN
American Society of Nephrology
JASN
Journal of the American Society of Nephrology
KIDNEY360
Accessing Our World From Every Angle
CJASN
Clinical Journal of the American Society of Nephrology

ASN Journal Disclosure Form

As per ASN journal policy, I have disclosed any financial relationships or commitments I have held in the past 36 months as included below. I have listed my Current Employer below to indicate there is a relationship requiring disclosure. If no relationship exists, my Current Employer is not listed.

Y. Brahmbhatt reports the following:
Employer: Novartis; Ownership Interest: Novartis; Research Funding: Novartis; Advisory or Leadership Role: Served on advisory boards as part of my job function at Novartis. I was not paid in addition for this.; and Other Interests or Relationships: ASN.

I understand that the information above will be published within the journal article, if accepted, and that failure to comply and/or to accurately and completely report the potential financial conflicts of interest could lead to the following: 1) Prior to publication, article rejection, or 2) Post-publication, sanctions ranging from, but not limited to, issuing a correction, reporting the inaccurate information to the authors' institution, banning authors from submitting work to ASN journals for varying lengths of time, and/or retraction of the published work.

Name: Yasmin G. Brahmbhatt
Manuscript ID: JASN-2026-000190
Manuscript Title: Atrasentan reduces proteinuria in patients with IgA nephropathy receiving sodium–glucose co-transporter-2 inhibitors: A randomized, double-blind, placebo-controlled crossover trial
Date of Completion: February 9, 2026
Disclosure Updated Date: February 9, 2026

ASN American Society of Nephrology | JASN Journal of the American Society of Nephrology | KIDNEY360 Accessing Our World From Every Angle | CJASN Clinical Journal of the American Society of Nephrology

ASN Journal Disclosure Form

As per ASN journal policy, I have disclosed any financial relationships or commitments I have held in the past 36 months as included below. I have listed my Current Employer below to indicate there is a relationship requiring disclosure. If no relationship exists, my Current Employer is not listed.

M. Dahlke reports the following:
Employer: Novartis Pharma AG; and Ownership Interest: Stock/stock options from Novartis Pharma AG, Sandoz and Alcon.

I understand that the information above will be published within the journal article, if accepted, and that failure to comply and/or to accurately and completely report the potential financial conflicts of interest could lead to the following: 1) Prior to publication, article rejection, or 2) Post-publication, sanctions ranging from, but not limited to, issuing a correction, reporting the inaccurate information to the authors' institution, banning authors from submitting work to ASN journals for varying lengths of time, and/or retraction of the published work.

Name: Marion Dahlke
Manuscript ID: JASN-2026-000190
Manuscript Title: Atrasentan reduces proteinuria in patients with IgA nephropathy receiving sodium–glucose co-transporter-2 inhibitors: A randomized, double-blind, placebo-controlled crossover trial
Date of Completion: February 13, 2026
Disclosure Updated Date: February 13, 2026

ASN American Society of Nephrology · JASN Journal of the American Society of Nephrology · KIDNEY360 Accessing Our World From Every Angle · CJASN Clinical Journal of the American Society of Nephrology

ASN Journal Disclosure Form

As per ASN journal policy, I have disclosed any financial relationships or commitments I have held in the past 36 months as included below. I have listed my Current Employer below to indicate there is a relationship requiring disclosure. If no relationship exists, my Current Employer is not listed.

M. Fu reports the following:
Employer: Hospital Sultanah Aminah Johor Bahru

I understand that the information above will be published within the journal article, if accepted, and that failure to comply and/or to accurately and completely report the potential financial conflicts of interest could lead to the following: 1) Prior to publication, article rejection, or 2) Post-publication, sanctions ranging from, but not limited to, issuing a correction, reporting the inaccurate information to the authors' institution, banning authors from submitting work to ASN journals for varying lengths of time, and/or retraction of the published work.

Name: Mei Sian Fu
Manuscript ID: JASN-2026-000190
Manuscript Title: Atrasentan reduces proteinuria in patients with IgA nephropathy receiving sodium–glucose co-transporter-2 inhibitors: A randomized, double-blind, placebo-controlled crossover trial
Date of Completion: February 11, 2026
Disclosure Updated Date: February 11, 2026

ASN American Society of Nephrology | JASN Journal of the American Society of Nephrology | KIDNEY360 Accessing Our World From Every Angle | CJASN Clinical Journal of the American Society of Nephrology

ASN Journal Disclosure Form

As per ASN journal policy, I have disclosed any financial relationships or commitments I have held in the past 36 months as included below. I have listed my Current Employer below to indicate there is a relationship requiring disclosure. If no relationship exists, my Current Employer is not listed.

J. Gorriz reports the following:
Employer: Department of Nephrology. Hospital Clinico Universitario; Consultancy: AstraZeneca, Bayer, Boehringer-Ingelheim and Novo Nordisk, CSL; Research Funding: Astrazeneca (The payments have been made to the INCLIVA research institute); Honoraria: Honoraria for giving talks: Boehringer-Ingelheim, Astrazeneca, Novonordisk, Novartis and Eli Lilly, Bayer.;; Advisory or Leadership Role: Astrazeneca, Boehringer-Ingelheim, Novonordisk, Bayer; and Speakers Bureau: Boehringer-Ingelheim, Astrazeneca, Novonordisk, Bayer.

I understand that the information above will be published within the journal article, if accepted, and that failure to comply and/or to accurately and completely report the potential financial conflicts of interest could lead to the following: 1) Prior to publication, article rejection, or 2) Post-publication, sanctions ranging from, but not limited to, issuing a correction, reporting the inaccurate information to the authors' institution, banning authors from submitting work to ASN journals for varying lengths of time, and/or retraction of the published work.

Name: Jose L. Gorriz
Manuscript ID: JASN-2026-000190R1
Manuscript Title: "Efficacy and Safety of Atrasentan in Patients with IgA Nephropathy Receiving Sodium–Glucose Co-Transporter-2 Inhibitors: A Placebo-Controlled, Crossover Trial."
Date of Completion: February 24, 2026
Disclosure Updated Date: February 15, 2026

ASN American Society of Nephrology | JASN Journal of the American Society of Nephrology | KIDNEY360 Accessing Our World From Every Angle | CJASN Clinical Journal of the American Society of Nephrology

ASN Journal Disclosure Form

As per ASN journal policy, I have disclosed any financial relationships or commitments I have held in the past 36 months as included below. I have listed my Current Employer below to indicate there is a relationship requiring disclosure. If no relationship exists, my Current Employer is not listed.

H. Heerspink reports the following:
Employer: University Medical Center Groningen; Consultancy: Ongoing consultancy agreements with AstraZeneca, Alexion, Alnylum, Bayer, Boehringer Ingelheim, Biocity Biopharmaceuticals, Dimerix, Eli-Lilly, Novartis, NovoNordisk, Otsuka, Roche, Travere Therapeutics; Research Funding: AstraZeneca, Bayer, Boehringer Ingelheim, and NovoNordisk research support (grant funding directed to employer); Honoraria: Lecture fees from AstraZeneca, Bayer, Eli-Lilly and NovoNordisk; and Speakers Bureau: AstraZeneca, Bayer, Novo Nordisk.

I understand that the information above will be published within the journal article, if accepted, and that failure to comply and/or to accurately and completely report the potential financial conflicts of interest could lead to the following: 1) Prior to publication, article rejection, or 2) Post-publication, sanctions ranging from, but not limited to, issuing a correction, reporting the inaccurate information to the authors' institution, banning authors from submitting work to ASN journals for varying lengths of time, and/or retraction of the published work.

Name: Hiddo Jan L. Heerspink
Manuscript ID: JASN-2026-000190
Manuscript Title: Atrasentan reduces proteinuria in patients with IgA nephropathy receiving sodium–glucose co-transporter-2 inhibitors: A randomized, double-blind, placebo-controlled crossover trial
Date of Completion: February 7, 2026
Disclosure Updated Date: February 7, 2026

ASN Journal Disclosure Form

As per ASN journal policy, I have disclosed any financial relationships or commitments I have held in the past 36 months as included below. I have listed my Current Employer below to indicate there is a relationship requiring disclosure. If no relationship exists, my Current Employer is not listed.

G. Housler has nothing to disclose.

I understand that the information above will be published within the journal article, if accepted, and that failure to comply and/or to accurately and completely report the potential financial conflicts of interest could lead to the following: 1) Prior to publication, article rejection, or 2) Post-publication, sanctions ranging from, but not limited to, issuing a correction, reporting the inaccurate information to the authors' institution, banning authors from submitting work to ASN journals for varying lengths of time, and/or retraction of the published work.

Name: Greggory Joseph Housler
Manuscript ID: JASN-2026-000190
Manuscript Title: Atrasentan reduces proteinuria in patients with IgA nephropathy receiving sodium–glucose co-transporter-2 inhibitors: A randomized, double-blind, placebo-controlled crossover trial
Date of Completion: February 9, 2026
Disclosure Updated Date: February 9, 2026

ASN
American Society of Nephrology
JASN
Journal of the American Society of Nephrology
KIDNEY360
Accessing Our World From Every Angle
CJASN
Clinical Journal of the American Society of Nephrology

ASN Journal Disclosure Form

As per ASN journal policy, I have disclosed any financial relationships or commitments I have held in the past 36 months as included below. I have listed my Current Employer below to indicate there is a relationship requiring disclosure. If no relationship exists, my Current Employer is not listed.

R. Jiao reports the following:
Employer: Novartis Pharmaceuticals Corporation; and Ownership Interest: Novartis Pharmaceuticals Corporation.

I understand that the information above will be published within the journal article, if accepted, and that failure to comply and/or to accurately and completely report the potential financial conflicts of interest could lead to the following: 1) Prior to publication, article rejection, or 2) Post-publication, sanctions ranging from, but not limited to, issuing a correction, reporting the inaccurate information to the authors' institution, banning authors from submitting work to ASN journals for varying lengths of time, and/or retraction of the published work.

Name: Rong Jiao
Manuscript ID: JASN-2026-000190
Manuscript Title: Atrasentan reduces proteinuria in patients with IgA nephropathy receiving sodium–glucose co-transporter-2 inhibitors: A randomized, double-blind, placebo-controlled crossover trial," submitted to the Journal of the American Society of Nephrology
Date of Completion: February 11, 2026
Disclosure Updated Date: February 11, 2026

ASN American Society of Nephrology
JASN Journal of the American Society of Nephrology
KIDNEY360 Accessing Our World From Every Angle
CJASN Clinical Journal of the American Society of Nephrology

ASN Journal Disclosure Form

As per ASN journal policy, I have disclosed any financial relationships or commitments I have held in the past 36 months as included below. I have listed my Current Employer below to indicate there is a relationship requiring disclosure. If no relationship exists, my Current Employer is not listed.

S. Kim reports the following:
Employer: Hallym University Sacred Heart Hospital; Consultancy: GSK; Alpine; AstraZeneca; Billy; Alexion; Bayer; Novatis; Novo Nordisk; CSL Vifor; Otsuka; Research Funding: Fibrogen; GSK; JW; Vera Therapeutics; Alpine; AstraZeneca; Billy; Alexion; Bayer; Roche; Novatis; Novo Nordisk; CSL Vifor; Otsuka; Honoraria: Alpine; AstraZeneca; Billy; Alexion; Bayer; Novatis; Novo Nordisk; CSL Vifor; and Advisory or Leadership Role: KSN; KSPD; KSHHD; FMC; Ventive; Alexion; Alpine.

I understand that the information above will be published within the journal article, if accepted, and that failure to comply and/or to accurately and completely report the potential financial conflicts of interest could lead to the following: 1) Prior to publication, article rejection, or 2) Post-publication, sanctions ranging from, but not limited to, issuing a correction, reporting the inaccurate information to the authors' institution, banning authors from submitting work to ASN journals for varying lengths of time, and/or retraction of the published work.

Name: Sung Gyun Kim
Manuscript ID: JASN-2026-000190
Manuscript Title: Atrasentan reduces proteinuria in patients with IgA nephropathy receiving sodium–glucose co-transporter-2 inhibitors: A randomized, double-blind, placebo-controlled crossover trial.
Date of Completion: February 15, 2026
Disclosure Updated Date: August 7, 2025

ASN American Society of Nephrology
JASN Journal of the American Society of Nephrology
KIDNEY360 Accessing Our World From Every Angle
CJASN Clinical Journal of the American Society of Nephrology

ASN Journal Disclosure Form

As per ASN journal policy, I have disclosed any financial relationships or commitments I have held in the past 36 months as included below. I have listed my Current Employer below to indicate there is a relationship requiring disclosure. If no relationship exists, my Current Employer is not listed.

S. Kotwal reports the following:
Employer: Spouse employed by Google; Consultancy: Dimerix Pharmaceuticals; Chinook/Novartis Pharmaceuticals, Amgen, Novo Nordisk, The Limbic; Ownership Interest: Google; Research Funding: The George Institute and its affiliated entities work with numerous health and pharmaceutical companies in the design, implementation and analyses of clinical research and clinical trials. It is possible that some of these companies have products relevant to the clinical space covered in this analysis, but Dr Kotwal is not aware of any possible conflicts arising from this work. Bayer supplies the study medication for my trial. NHMRC and MRFF funding.; Honoraria: Novartis. Novo Nordisk; and Advisory or Leadership Role: Dimerix Pharmaceuticals and Chinook/Novartis Pharmaceuticals (steering committee member - paid); ANZDATA Advisory Committee (unpaid).

I understand that the information above will be published within the journal article, if accepted, and that failure to comply and/or to accurately and completely report the potential financial conflicts of interest could lead to the following: 1) Prior to publication, article rejection, or 2) Post-publication, sanctions ranging from, but not limited to, issuing a correction, reporting the inaccurate information to the authors' institution, banning authors from submitting work to ASN journals for varying lengths of time, and/or retraction of the published work.

Name: Sradha S. Kotwal
Manuscript ID: JASN-2026-000190
Manuscript Title: Atrasentan reduces proteinuria in patients with IgA nephropathy receiving sodium–glucose co-transporter-2 inhibitors: A randomized, double-blind, placebo-controlled crossover trial
Date of Completion: February 8, 2026
Disclosure Updated Date: November 13, 2025

ASN American Society of Nephrology
JASN Journal of the American Society of Nephrology
KIDNEY360 Accessing Our World From Every Angle
CJASN Clinical Journal of the American Society of Nephrology

ASN Journal Disclosure Form

As per ASN journal policy, I have disclosed any financial relationships or commitments I have held in the past 36 months as included below. I have listed my Current Employer below to indicate there is a relationship requiring disclosure. If no relationship exists, my Current Employer is not listed.

S. Lim reports the following:
Employer: University of Malaya

I understand that the information above will be published within the journal article, if accepted, and that failure to comply and/or to accurately and completely report the potential financial conflicts of interest could lead to the following: 1) Prior to publication, article rejection, or 2) Post-publication, sanctions ranging from, but not limited to, issuing a correction, reporting the inaccurate information to the authors' institution, banning authors from submitting work to ASN journals for varying lengths of time, and/or retraction of the published work.

Name: Soo Kun Lim
Manuscript ID: JASN-2026-000190
Manuscript Title: Atrasentan reduces proteinuria in patients with IgA nephropathy receiving sodium–glucose co-transporter-2 inhibitors: A randomized, double-blind, placebo-controlled crossover trial,
Date of Completion: February 17, 2026
Disclosure Updated Date: February 17, 2026

ASN American Society of Nephrology
JASN Journal of the American Society of Nephrology
KIDNEY360 Accessing Our World From Every Angle
CJASN Clinical Journal of the American Society of Nephrology

ASN Journal Disclosure Form

As per ASN journal policy, I have disclosed any financial relationships or commitments I have held in the past 36 months as included below. I have listed my Current Employer below to indicate there is a relationship requiring disclosure. If no relationship exists, my Current Employer is not listed.

A. Lodha reports the following:
Employer: Novartis Pharmaceuticals Corporation; and Ownership Interest: Novartis Pharmaceuticals Corporation (Stocks and Restricted Stock Awards).

I understand that the information above will be published within the journal article, if accepted, and that failure to comply and/or to accurately and completely report the potential financial conflicts of interest could lead to the following: 1) Prior to publication, article rejection, or 2) Post-publication, sanctions ranging from, but not limited to, issuing a correction, reporting the inaccurate information to the authors' institution, banning authors from submitting work to ASN journals for varying lengths of time, and/or retraction of the published work.

Name: Amit Lodha
Manuscript ID: JASN-2026-000190
Manuscript Title: Atrasentan Reduces Proteinuria in Patients with IgA Nephropathy Receiving Sodium-Glucose Co-Transporter-2 Inhibitors: A Randomized, Double-Blind, Placebo-Controlled Crossover Trial
Date of Completion: February 17, 2026
Disclosure Updated Date: February 17, 2026

ASN American Society of Nephrology
JASN Journal of the American Society of Nephrology
KIDNEY360 Accessing Our World From Every Angle
CJASN Clinical Journal of the American Society of Nephrology

ASN Journal Disclosure Form

As per ASN journal policy, I have disclosed any financial relationships or commitments I have held in the past 36 months as included below. I have listed my Current Employer below to indicate there is a relationship requiring disclosure. If no relationship exists, my Current Employer is not listed.

G. Mastroianni-Kirsztajn has nothing to disclose.

I understand that the information above will be published within the journal article, if accepted, and that failure to comply and/or to accurately and completely report the potential financial conflicts of interest could lead to the following: 1) Prior to publication, article rejection, or 2) Post-publication, sanctions ranging from, but not limited to, issuing a correction, reporting the inaccurate information to the authors' institution, banning authors from submitting work to ASN journals for varying lengths of time, and/or retraction of the published work.

Name: Gianna Mastroianni-Kirsztajn
Manuscript ID: JASN-2026-000190
Manuscript Title: Atrasentan reduces proteinuria in patients with IgA nephropathy receiving sodium–glucose co-transporter-2 inhibitors: A randomized, double-blind, placebo-controlled crossover trial
Date of Completion: February 6, 2026
Disclosure Updated Date: February 6, 2026

ASN American Society of Nephrology
JASN Journal of the American Society of Nephrology
KIDNEY360 Accessing Our World From Every Angle
CJASN Clinical Journal of the American Society of Nephrology

ASN Journal Disclosure Form

As per ASN journal policy, I have disclosed any financial relationships or commitments I have held in the past 36 months as included below. I have listed my Current Employer below to indicate there is a relationship requiring disclosure. If no relationship exists, my Current Employer is not listed.

A. Mottl reports the following:
Employer: University of North Carolina at Chapel Hill; Consultancy: Bayer, Chinook, Otsuka, Novartis, Vera; Research Funding: Alexion, Bayer, Boehringer Ingelheim, Chinook, Duke Clinical Research Institute, Novartis, University of Pennsylvania, Vera; Honoraria: UpToDate; and Advisory or Leadership Role: Bayer, Chinook, Novartis, Otsuka, Vera.

I understand that the information above will be published within the journal article, if accepted, and that failure to comply and/or to accurately and completely report the potential financial conflicts of interest could lead to the following: 1) Prior to publication, article rejection, or 2) Post-publication, sanctions ranging from, but not limited to, issuing a correction, reporting the inaccurate information to the authors' institution, banning authors from submitting work to ASN journals for varying lengths of time, and/or retraction of the published work.

Name: Amy K. Mottl
Manuscript ID: JASN-2026-000190
Manuscript Title: Atrasentan reduces proteinuria in patients with IgA nephropathy receiving sodium–glucose co-transporter-2 inhibitors: A randomized, double-blind, placebo-controlled crossover trial
Date of Completion: February 10, 2026
Disclosure Updated Date: February 10, 2026

ASN American Society of Nephrology
JASN Journal of the American Society of Nephrology
KIDNEY360 Accessing Our World From Every Angle
CJASN Clinical Journal of the American Society of Nephrology

ASN Journal Disclosure Form

As per ASN journal policy, I have disclosed any financial relationships or commitments I have held in the past 36 months as included below. I have listed my Current Employer below to indicate there is a relationship requiring disclosure. If no relationship exists, my Current Employer is not listed.

J. De resende barros neto has nothing to disclose.

I understand that the information above will be published within the journal article, if accepted, and that failure to comply and/or to accurately and completely report the potential financial conflicts of interest could lead to the following: 1) Prior to publication, article rejection, or 2) Post-publication, sanctions ranging from, but not limited to, issuing a correction, reporting the inaccurate information to the authors' institution, banning authors from submitting work to ASN journals for varying lengths of time, and/or retraction of the published work.

Name: José De resende barros neto
Manuscript ID: JASN-2026-000190
Manuscript Title: "Atrasentan reduces proteinuria in patients with IgA nephropathy receiving sodium–glucose co-transporter-2 inhibitors: A randomized, double-blind, placebo-controlled crossover trial."
Date of Completion: February 16, 2026
Disclosure Updated Date: February 16, 2026

ASN American Society of Nephrology
JASN Journal of the American Society of Nephrology
KIDNEY360 Accessing Our World From Every Angle
CJASN Clinical Journal of the American Society of Nephrology

ASN Journal Disclosure Form

As per ASN journal policy, I have disclosed any financial relationships or commitments I have held in the past 36 months as included below. I have listed my Current Employer below to indicate there is a relationship requiring disclosure. If no relationship exists, my Current Employer is not listed.

I. Noronha reports the following:
Employer: University of Sao Paulo; Consultancy: Roche, Novartis, ProKidney, Vertex, Alexion; Honoraria: AstraZeneca, Roche, GSK, Alexion; and Advisory or Leadership Role: International Society of Nephrology; International Society of Transplantation; Brazilian Society of Nephrology; Brazilian Society for Organ Transplantation; Brazilian Journal of Nephrology; Jornal Brasileiro de Transplantes.

I understand that the information above will be published within the journal article, if accepted, and that failure to comply and/or to accurately and completely report the potential financial conflicts of interest could lead to the following: 1) Prior to publication, article rejection, or 2) Post-publication, sanctions ranging from, but not limited to, issuing a correction, reporting the inaccurate information to the authors' institution, banning authors from submitting work to ASN journals for varying lengths of time, and/or retraction of the published work.

Name: Irene L. Noronha
Manuscript ID: JASN-2026-000190R1
Manuscript Title: "Efficacy and Safety of Atrasentan in Patients with IgA Nephropathy Receiving Sodium–Glucose Co-Transporter-2 Inhibitors: A Placebo-Controlled, Crossover Trial.
Date of Completion: February 24, 2026
Disclosure Updated Date: February 24, 2026

ASN
American Society of Nephrology
JASN
Journal of the American Society of Nephrology
KIDNEY360
Accessing Our World From Every Angle
CJASN
Clinical Journal of the American Society of Nephrology

ASN Journal Disclosure Form

As per ASN journal policy, I have disclosed any financial relationships or commitments I have held in the past 36 months as included below. I have listed my Current Employer below to indicate there is a relationship requiring disclosure. If no relationship exists, my Current Employer is not listed.

J. Ryan reports the following:
Employer: Monash Health; and Honoraria: CSL Vifor; Novartis, Sobi.

I understand that the information above will be published within the journal article, if accepted, and that failure to comply and/or to accurately and completely report the potential financial conflicts of interest could lead to the following: 1) Prior to publication, article rejection, or 2) Post-publication, sanctions ranging from, but not limited to, issuing a correction, reporting the inaccurate information to the authors' institution, banning authors from submitting work to ASN journals for varying lengths of time, and/or retraction of the published work.

Name: Jessica Ryan
Manuscript ID: JASN-2026-000190
Manuscript Title: Atrasentan reduces proteinuria in patients with IgA nephropathy receiving sodium–glucose co-transporter-2 inhibitors: A randomized, double-blind, placebo-controlled crossover trial
Date of Completion: February 11, 2026
Disclosure Updated Date: February 8, 2026
