# Supplementary material for: Efficacy and Safety of Atrasentan in Patients with IgA Nephropathy Receiving Sodium-Glucose Cotransporter 2 Inhibitors: Placebo-Controlled, Crossover Trial
Source: J Am Soc Nephrol. 2026 Mar 29;37(9):2004–15. doi: 10.1681/ASN.0000001076 (PMC13567876; doi:10.1681/ASN.0000001076)
Supplement: Supplementary file 2 [file jasn-37-2004-s002.pdf]

Supplemental Material

The authors have provided this appendix to give readers additional information about this work.

Supplement to: Hiddo J.L. Heerspink, Irene L. Noronha, Jose Luis Górriz *et al.* Efficacy and Safety of Atrasentan in Patients with IgA Nephropathy Receiving Sodium-Glucose Cotransporter 2 Inhibitors: Placebo-Controlled, Crossover Trial

Table of contents:

List of ASSIST steering committee members 2

List of ASSIST principal investigators[a] 3

Supplemental Methods 4

Eligibility Criteria...4

Supplementary analyses for the primary end point 7

Supplemental Table 1. Methodology for supplementary analyses of the primary end point .7

Supplemental Results 8

Supplemental Table 2. Pre-specified supplementary analysis 1 of the primary end point: change from baseline to week 12 in 24-hour UPCR[a] 8

Supplemental Table 3. Pre-specified supplementary analysis 2 of the primary end point: change from baseline to week 12 in 24-hour UPCR[a] 9

Supplemental Table 4. Pre-specified supplementary analysis 3 of the primary end point: change from baseline to week 12 in 24-hour UPCR[a] 10

Supplemental Table 5. Geometric mean ratio of 24-hour UPCR[a] at week 12 versus baseline within each treatment period 11

Supplemental Table 6. Most common treatment-emergent adverse events (TEAEs; >5% of patients)...12

Supplemental Figure 1. eGFR by visit (safety analysis set)..13

Statistical Analysis Plan

List of ASSIST steering committee members

Country	Steering Committee Member
Australia	Sradha Kotwal
Brazil	Irene Noronha
Malaysia	Soo Kun Lim
Netherlands	Hiddo J.L. Heerspink[a]
South Korea	Sung-Gyun Kim
Spain	Jose Luis Górriz
United Kingdom	Jonathan Barratt
United States of America	Amy Mottl[a]

[a]Steering committee co-chair.

List of ASSIST principal investigators[a]

Country	Principal Investigator
Australia	• Sradha Kotwal[b]<br>• Eugenia Pedagogos<br>• Jessica Ryan
Brazil	• Elizete Keitel<br>• Gianna Kirsztajn<br>• José de Resende Barros Neto<br>• Irene Noronha[b]
Malaysia	• Abdul Halim Abdul Gafor<br>• Mei Sian Fu<br>• Soo Kun Lim[b]<br>• Kah Mean Thong
South Korea	• Won Suk An<br>• Jungho Shin
Spain	• Secundino Cigarran Guldris<br>• Francisco Gonzalez<br>• Jose Luis Górriz[b]<br>• Eva Marquez Mosquera<br>• Judith Fatima Martins Munoz<br>• Mercedes Salgueira Lazo
United States of America	• Lesley Inker<br>• Amy Mottl[c]

[a]The table lists principal investigators who randomized at least one patient; names are ordered alphabetically by country.
[b]ASSIST steering committee member.
[c]ASSIST steering committee co-chair.

Supplemental Methods

Eligibility Criteria

Inclusion Criteria

1. Legal adults (per local and country specifications) ≥18 years of age at the time of signing the informed consent form prior to initiation of any study-specific activities/procedures
2. Biopsy-proven immunoglobulin A (IgA) nephropathy (IgAN) that, in the opinion of the investigator, is not due to secondary causes
    a. Biopsy could have occurred at any point in time prior to study
    b. A diagnostic report must have been available for review by the Sponsor or designee
3. Receiving a maximally tolerated and stable dose of a renin–angiotensin system inhibitor (RASi) for at least 12 weeks prior to screening. Investigator discretion should have been used in determining maximally tolerated and optimized dose
4. Estimated glomerular filtration rate (eGFR) ≥30 ml/min/1.73 m^2 at screening based on the 2021 CKD-EPI (Chronic Kidney Disease Epidemiology Collaboration) equation (https://www.kidney.org/professionals/kdoqi/gfr_calculator)
5. Willing to abide with highly effective forms of contraception, throughout the study and for up to 1 month afterward. In women of child-bearing potential, use of hormonal contraceptive agents must have been started at least 1 month prior to baseline
6. Provided informed consent and agreed to comply with all study visits and study procedures

Inclusion criteria for sodium glucose co-transporter-2 inhibitor (*SGLT2i) stable patients*

7. Receiving treatment with a SGLT2i at a stable dose for at least 8 weeks prior to screening
8. 24-hour total urine protein of >0.5 g/day

Inclusion criteria for SGLT2i run-in patients

9. 24-hour total urine protein of >0.85 g/day at screening
10. Completed an 8-week run-in period with a stable and well-tolerated dose of SGLT2i

11. 24-hour total urine protein of >0.5 g/day confirmed at the run-in week 8 visit
12. eGFR of ≥30 ml/min/1.73 m^2 (based on the CKD-EPI equation) at the run-in week 8 visit

Patients who did not complete the 8-week run-in period, or were unable to fulfil the last two criteria during the run-in period, were considered unsuccessful in completing the run-in period.

Exclusion Criteria

1. Concurrent diagnosis of another cause of chronic kidney disease including diabetic kidney disease or another primary glomerulopathy
2. Clinical suspicion of rapidly progressive glomerulonephritis based on the Kidney Disease: Improving Global Outcomes (KDIGO) guidelines or clinical suspicion of Henoch-Schonlein Purpura (IgA vasculitis)
3. Clinical diagnosis of nephrotic syndrome
4. Diagnosis of type 1 diabetes
5. B-type natriuretic peptide value of >200 pg/ml at screening
6. Platelet count <80,000/μl at screening
7. History of organ transplantation (patients with history of corneal transplant were excluded)
8. Use of systemic immunosuppressant medications including systemic corticosteroids (eg prednisone, prednisolone, budesonide, etc) mycophenolate, azathioprine, cyclosporine, tacrolimus, etc; use of herbs such as *Tripterygium Wilfordii* Hook F, *Caulis sinomenii*, and *Sinomenium acutum*; for >2 weeks in the past 3 months. Use of rituximab within the past 6 months
9. Confirmed blood pressure >150 mmHg systolic or >95 mmHg diastolic based on a blood pressure measurement obtained at screening
10. Known history of heart failure or prior hospital admissions for conditions relating to fluid overload including, but not limited to, pulmonary edema, uncontrolled peripheral edema, pleural effusion, or ascites that in the opinion of the Investigator or Sponsor's Medical Lead (or designee) might confound the results of the study or pose additional risk to the patient by their participation in the study
11. Known history of clinically significant liver disease or transaminase or bilirubin values more than twice the upper limit of normal. Patients with treated hepatitis C could be considered for inclusion into the study upon consultation with the Sponsor's Medical Lead (or designee)

12. Hemoglobin below 9 g/dL at screening or prior history of blood transfusion for anemia within 3 months of screening
13. History of malignancy unless cancer free for at least 5 years or nonmelanoma skin cancer that was completely resected. A patient with curatively treated cervical carcinoma *in situ* was eligible for this study
14. Pregnancy, breast-feeding, or intent to become pregnant during the study period and at least 1 month afterward for females
15. Intent to father a child or donate sperm during the study period and at least 1 month afterward for males
16. Had received any investigational agent or approved treatment for IgAN (other than a RASi and SGT2i) within 1 month (or five half-lives of the agent, whichever is longer) prior to screening. If the investigational agent was a cytotoxic or immunosuppressive agent, then this washout period was 6 months
17. Concurrent clinically significant, unstable, or uncontrolled cardiovascular, pulmonary, hepatic, renal, gastrointestinal, genitourinary, hematological, coagulation, immunological, endocrine/metabolic, or other medical disorder that, in the opinion of the Investigator or Sponsor's Medical Lead (or designee), might confound the results of the study or pose additional risk to the patient by their participation in the study
18. History of an alcohol or illicit drug-related disorder within the past 1 year

Supplemental analyses for the primary end point

To assess the robustness of the results of the primary analysis and the impact of intercurrent events, supplementary analysis 1 and 2 with different handling strategies of intercurrent events were planned.

Given that the primary analysis conducted multiple imputations over missing end points, supplementary analysis 3 was planned on observed datasets only without any imputation, applying the same handling strategy of intercurrent events.

Supplemental Table 1. Methodology for supplementary analyses of the primary end point

		Analysis for the primary end point			
		**Primary analysis**	**Supplementary analysis 1**	**Supplementary analysis 2**	**Supplementary analysis 3**
	**Handling of missing end points**	Multiple Imputation	Multiple Imputation	Multiple Imputation	No imputation
	**Handling strategies of intercurrent events**				
1	Study drug discontinuation for any reason	Treatment policy strategy	*	*	*
2	Initiation of maintenance dialysis or kidney transplantation	Treatment policy strategy	*	Hypothetical strategy	*
3	Dose modification or discontinuation of background therapy (RASi/SGLT2i)	Treatment policy strategy	*	*	*
4	Initiation of prohibited or restricted concomitant medication	Treatment policy strategy	*	Hypothetical strategy	*
5	Initiation of an investigational or approved product for the treatment of IgAN (other than RASi and SGLT2i)	Hypothetical strategy	Treatment policy strategy	*	*

A star (*) in cells for supplementary analyses indicates that this cell entry is identical to the one for the main analysis (primary end point analysis).

IgAN, immunoglobulin A nephropathy; RASi, renin–angiotensin system inhibitor; SGTL2i, sodium-glucose cotransporter 2 inhibitor.

Supplemental Results

Supplemental Table 2. Pre-specified supplementary analysis 1 of the primary end point: change from baseline to week 12 in 24-hour UPCR[a]

	Atrasentan (*N* = 54)[c]	Placebo (*N* = 54)[c]	Within-individual difference (atrasentan–placebo)[b]
n[c]	50	49	46
Change from baseline-natural log			
LSMean (SE)	−0.37 (0.06)	−0.08 (0.06)	−0.29 (0.09)
95% CI	−0.49 to −0.25	−0.20 to 0.05	−0.46 to −0.13
Geometric mean % change from baseline			
LSMean	−30.7	−7.2	−25.3
95% CI	−38.5 to −21.9	−17.7 to 4.7	−36.8 to −11.7
P value			0.0006[d]

[a]UPCR in a logarithmic scale change from baseline (within each treatment period) was analyzed based on a mixed model with sequence, period, and treatment as fixed-effect factors, baseline natural log UPCR and the randomization stratification factor of SGLT2i status (stable vs run-in) as covariates, and with a random effect of participants nested within sequences. Rubin's rules were applied to the estimates from multiple imputation.

[b]Within-individual difference: the difference in atrasentan and placebo was derived from a within-participant comparison.

[c]*N*: participants in ITT Analysis set. *n*: number of participants with non-missing observation. Geometric mean % change from baseline = −100*(1−exp(change from baseline-natural log LSmean)). ICE handling strategy: treatment policy strategy applied to all five ICEs.

[d]Indicates two-sided nominal *P* value <0.05.

CI, confidence interval; ICE, intercurrent event; ITT, intention-to-treat; LS, least squares; SE, standard error; SGTL2i, sodium-glucose cotransporter 2 inhibitor; UPCR, urinary protein-to-creatinine ratio.

Supplemental Table 3. Pre-specified supplementary analysis 2 of the primary end point: change from baseline to week 12 in 24-hour UPCR[a]

	Atrasentan (*N* = 54)[c]	Placebo (*N* = 54)[c]	Within-individual difference (atrasentan–placebo)[b]
n[c]	50	49	46
Change from baseline-natural log			
LSMean (SE)	−0.37 (0.06)	−0.09 (0.06)	−0.28 (0.09)
95% CI	−0.49 to −0.25	−0.21 to 0.04	−0.45 to –0.11
Geometric mean % change from baseline			
LSMean	−30.6	−8.2	−24.4
95% CI	−38.4 to −21.8	−18.5 to 3.5	−36.0 to −10.7
P value			0.0010[d]

[a]UPCR in a logarithmic scale change from baseline (within each treatment period) was analyzed based on a mixed model with sequence, period, and treatment as fixed-effect factors, baseline natural log UPCR and the randomization stratification factor of SGLT2i status (stable vs run-in) as covariates, and with a random effect of participants nested within sequences. Rubin's rules were applied to the estimates from multiple imputation.

[b]Within-individual difference: the difference in atrasentan and placebo was derived from a within-participant comparison.

[c]*N*: participants in ITT Analysis set. *n*: number of participants with non-missing observation. Geometric mean % change from baseline = −100*(1−exp(change from baseline-natural log LSmean)). ICE handling strategy: treatment policy strategy applied to "Study drug discontinuation for any reason" and "Dose modification or discontinuation of background therapy (RASi/SGLT2i)", hypothetical strategy applied to the rest three ICEs.

[d]Indicates two-sided nominal *P* value <0.05.

CI, confidence interval; ICE, intercurrent event; ITT, intention-to-treat; LS, least squares; SE, standard error; SGTL2i, sodium-glucose cotransporter 2 inhibitor; UPCR, urinary protein-to-creatinine ratio.

Supplemental Table 4. Pre-specified supplementary analysis 3 of the primary end point: change from baseline to week 12 in 24-hour UPCR[a]

	Atrasentan (*N* = 54)[c]	Placebo (*N* = 54)[c]	Within-individual difference (atrasentan–placebo)[b]
n[c]	50	49	46
Change from baseline-natural log			
LSMean (SE)	−0.37 (0.06)	−0.07 (0.06)	−0.30 (0.09)
95% CI	−0.50 to −0.25	−0.20 to 0.05	−0.47 to −0.13
Geometric mean % change from baseline			
LSMean	−31.2	−7.1	−26.0
95% CI	−39.1 to −22.4	−17.8 to 5.0	−37.7 to −12.1
P value			0.0008[d]

[a]UPCR in a logarithmic scale change from baseline (within each treatment period) was analyzed based on a mixed model with sequence, period, and treatment as fixed-effect factors, baseline natural log UPCR and the randomization stratification factor of SGLT2i status (stable vs run-in) as covariates, and with a random effect of participants nested within sequences

[b]Within-individual difference: the difference in atrasentan and placebo was derived from a within-participant comparison.

[c]*N*: participants in ITT Analysis set. *n*: number of participants with non-missing observation. Geometric mean % change from baseline = −100*(1−exp(change from baseline-natural log LSmean)). ICE handling strategy: hypothetical strategy applied to “Initiation of an investigational or approved product for the treatment of IgAN (other than RASi and SGLT2i)”, treatment policy strategy applied to the rest four ICEs.

[d]Indicates 2-sided nominal *P* value <0.05.

CI, confidence interval; ICE, intercurrent event; ITT, intention-to-treat; LS, least squares; SE, standard error; SGTL2i, sodium-glucose cotransporter 2 inhibitor; UPCR, urinary protein-to-creatinine ratio.

Supplemental Table 5. Geometric mean ratio of 24-hour UPCR[a] at week 12 versus baseline within each treatment period

	Treatment period 1	Treatment period 2	Within-individual ratio[b] (atrasentan vs placebo)
**Atrasentan-placebo (*N* = 27)**			
Number of patients[c]	25	24	22
Geometric adjusted mean (95% CI) of ratio[d]	0.7 (0.6 to 0.8)	0.9 (0.7 to 1.0)	0.8 (0.6 to 1.0)
**Placebo-atrasentan (*N* = 27)**			
Number of patients[c]	25	25	24
Geometric adjusted mean (95% CI) of ratio[d]	1.0 (0.8 to 1.2)	0.7 (0.6 to 0.8)	0.7 (0.6 to 0.9)

[a]By treatment sequence, UPCR in a logarithmic scale change from baseline (within each treatment period) was analyzed based on mixed model treatment as fixed-effect factors, and baseline natural log UPCR and the randomization stratification factor of SGLT2i status (stable vs run-in) as covariates, with a random effect. Rubin's rules were applied to the estimates from multiple imputation.

[b]The ratio of atrasentan vs placebo is derived from a within-participant comparison.

[c]Number of participants with non-missing observations. Intercurrent event handing strategy: hypothetical strategy applied to "Initiation of an investigational or approved product for the treatment of IgAN (other than RASi and SGLT2i)", treatment policy strategy applied to the rest four intercurrent events.

[d]At each treatment period, the geometric adjusted mean and 95% CI for the ratio of 24-hour UPCR at week 12 versus baseline 24-hour UPCR are estimated by treatment sequence.

CI, confidence interval; IgAN, immunoglobulin A nephropathy; RASi, renin–angiotensin system inhibitors; SGLT2i, sodium–glucose co-transporter-2 inhibitors; UPCR, urinary protein-to-creatinine ratio.

Supplemental Table 6. Most common treatment-emergent adverse events (TEAEs; >5% of patients)

TEAE, *n* (%)	Treatment period 1		Treatment period 2	
	Atrasentan (*N* = 27)	Placebo (*N* = 27)	Placebo (*N* = 27)	Atrasentan (*N* = 27)
Upper respiratory tract infection	4 (15)	0	3 (11)	2 (7)
Fluid retention	3 (11)	0	3 (11)	1 (4)
Influenza	2 (7)	1 (4)	1 (4)	0
Back pain	0	2 (7)	0	2 (7)
Dizziness	2 (7)	0	0	1 (4)
Hypertension	0	2 (7)	1 (4)	2 (7)
Nasopharyngitis	0	2 (7)	2 (7)	1 (4)
Viral infection	2 (7)	0	0	0
Headache	0	1 (4)	2 (7)	2 (7)
Hypotension	1 (4)	1 (4)	3 (11)	1 (4)
Cough	1 (4)	1 (4)	2 (7)	0
Gastroenteritis	1 (4)	1 (4)	2 (7)	0

A participant with multiple occurrences for an AE was only counted once. TEAEs are defined as AEs that first occurred or worsened on or after the first dose of treatment in each randomized treatment period and within 30 days after the last dose of treatment in each period. MedDRA version 28.1 was used for the AE reporting.

AE, adverse event; TEAE, treatment-emergent adverse event.

Supplemental Figure 1. eGFR by visit (safety analysis set)

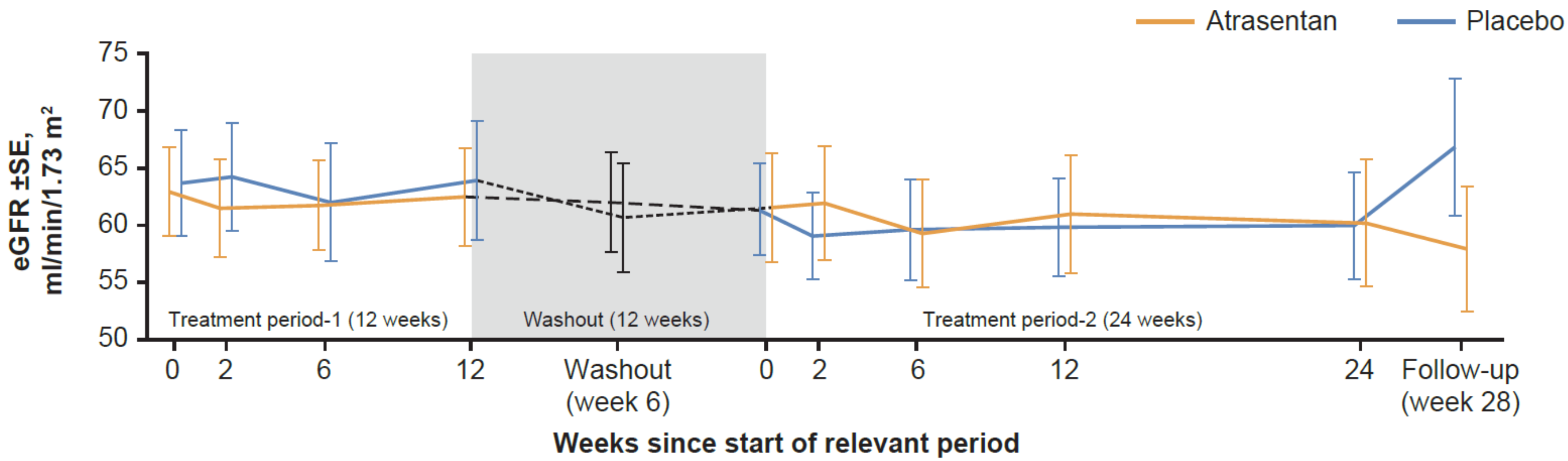

Number of patients

Atrasentan-to-placebo	27	26	26	27	25	27	26	26	26	25	17
Placebo-to-atrasentan	27	26	25	27	27	27	26	27	27	26	24

eGFR, estimated glomerular filtration rate.

Clinical Development

EXV811/Atrasentan®

CEXV811A12201

A Randomized, Double-blind, Placebo-controlled, Crossover Study of Atrasentan in Subjects with IgA Nephropathy on Sodium-glucose Cotransporter-2 Inhibitors (SGLT2i) (The ASSIST Study)

Statistical Analysis Plan (SAP)

Document type: SAP Documentation

Document status: Amendment 2.0

Release date: 31-Oct-2025

Number of pages: 48

Property of Novartis

Confidential

May not be used, divulged, published or otherwise disclosed

without the consent of Novartis

Template Version 4.0, Effective from 23-Apr-2021

Table of Contents

Table of Contents 2
List of tables 4
1 Introduction 8
2 Study Design 8
2.1 Description 8
2.2 Treatment Assignment and Blinding 9
2.3 Study Objectives and Endpoints 9
2.4 Sample Size 11
2.5 Schedule of analyses 11
2.5.1 Final Analysis 11
2.5.2 Multiple Comparisons and Type I Error Control 11
3 Definitions, Derivations, and Data-Handling Conventions 11
3.1 Analysis Sets 11
3.1.1 Screened Analysis Set 11
3.1.2 Intent-to-Treat Analysis Set 11
3.1.3 Run-In Analysis Set 12
3.1.4 Safety Analysis Set 12
3.1.5 Pharmacokinetic Analysis Set 12
[redacted] 12
12
3.2 Subgroups 12
3.3 Study Date Related Definitions 12
3.3.1 Date of First and Last Dose of Treatment 12
3.3.2 Date of Last Contact 13
3.3.3 Study Day 13
3.3.4 Baseline 13
3.3.5 Post-Baseline 14
3.4 Data Handling Conventions 15
3.4.1 Missing Data 15
3.4.2 Data Transformations and Conventions 17
3.4.3 Classification of prohibited/restricted medications 18
3.5 Analysis Conventions 18
3.5.1 Study Visits 18
3.5.2 General Statistical Methods 19
3.6 Other General Definitions 20

3.6.1 Run-in period 20
3.6.2 Treatment period 1 20
3.6.3 Washout period 20
3.6.4 Treatment period 2 20
3.6.5 Safety follow-up period....................20
3.6.6 On-treatment values 20
3.6.7 Last post-treatment values....................21
3.6.8 Data for safety reporting 21
4 Subject Disposition and Enrollment....................21
4.1 Disposition....................21
4.2 Enrollment and Randomization 22
4.3 Protocol Deviations 22
4.4 Medical History 22
5 Demographics and Baseline Characteristics 22
6 Study Treatment Exposure and Compliance 24
7 Analysis of Efficacy 25
7.1 Primary Endpoint....................25
7.1.1 Primary estimand 26
7.1.2 Sensitivity and Supportive Analyses....................28
7.2 Secondary Endpoint....................30
7.2.1 Secondary estimand 31
7.2.2 Sensitivity and Supportive Analyses....................32
7.3 Exploratory Endpoints....................32
7.3.1 eGFR in Treatment Period 2 32
CCI [redacted]33
33
8 Safety....................33
8.1 Adverse Events 34
8.1.1 Treatment-Emergent AEs and Deaths....................34
8.1.2 Adverse Events of Special Interest 36
8.2 Prior, Baseline, Concomitant, and Subsequent Medications....................37
8.2.1 Concomitant Medications of Special Interest 38
8.3 Clinical Laboratory Data 38
8.4 Body Weight and Vital Signs 40
8.5 Electrocardiograms....................41
8.6 Other Safety Measures....................41

9 Pharmacokinetics....41
10 Appendices....41
10.1 Appendix 1 Clinical Laboratory Tests....41
10.2 Appendix 2 List of Abbreviations....43
10.3 Appendix 3 Classification Algorithm....46
11 References....48

List of tables

Table 1: Start and End Date Imputation....16
Table 2: Visit Windows....19
Table 3: Handling Strategy for Intercurrent Events....30
Table 4: Classification of treatment-emergent AEs....34
Table 5: Adverse Events of Special Interest....36
Table 6: Criteria for Potential Clinical Significance....40
Table 7: Classification Algorithm....46
Table 8: Prednisone Equivalent Conversion....47

Document History – Changes compared to previous final version of SAP

Date	Time point	Reason for update	Outcome for update	Section and title impacted (Current)
29-Jul-2024	Prior to FPFV	Creation of final version	N/A – First version	NA
20-May-2025	After protocol amendment 02 (v03)	SMC meetings will be canceled	Remove Section on Safety Monitoring Committee	Section 2.5.1
		FMV urine will be added for baseline characteristics summary	Add baseline of FMV Urine	Section 3.3.4
		Will summarize AE during run-in period	Start and end date imputation on run-in period is added	Section 3.4.1.2
		Will define run-in period, treatment period 1, washout period and treatment period 2	Definitions on study period and data for safety reporting were added	Section 3.6
		Summarize subcategories of SLGT2i stable subjects and SGLT2i run-in subjects	Detailed subcategories were added	Section 4
		Will summarize demographics and baseline characteristics in two tables	Category order was reorganized and preferred units added	Section 5
		Uncertainty (%) should be < 20%	Definition of uncertainty was added	Section 6
		Clarify methodology details	A description paragraph was added	Section 7.1

Date	Time point	Reason for update	Outcome for update	Section and title impacted (Current)
		Protocol amendment updated handling of intercurrent events	Handling of intercurrent events was updated	Section 7.1.1
		Protocol amendment added sensitivity analysis using treatment policy to handle all intercurrent events	Sensitivity analysis using treatment policy to handle all intercurrent events was added	Section 7.1.2
		The study has two treatment periods	Classification of TEAE for two treatment periods were added	Section 8.1.1
		Will summarize AE over run-in period and washout period	Definitions of AE over run-in period and washout period were added	Section 8.1.1
		Will summarize ALT or AST + Total Bilirubin based on peak values	Criteria on peak values was added	Section 8.3
31-Oct-2025	Before Final DBL	Select prohibited/restricted medication as most important intercurrent events for analysis	Classification of Prohibited/Restricted medication	Section 3.4.3, Appendix 3
		Add imputation rule of procedure dates	Imputation rule of procedure dates	Section 3.4.1.2
		Revise start date imputation when Run-in Period, Treatment Period 1, or Treatment Period 2 start in the same year	Imputation of AE/medication start date	Section 3.4.1.2
		Add a time window for selected assessments	Baseline value	Section 3.3.4, 3.4.1.1

Date	Time point	Reason for update	Outcome for update	Section and title impacted (Current)
		Move duration of disease to Section 5	Duration of disease	Section 4.4
		Add and remove baseline characteristics to be summarized	Baseline characteristics	Section 5
		Evaluate period effect	An analysis table and a summary table were added	Section 7.1
		Display intercurrent events	Two listings were added	Section 7.1.1
		Model-based analysis on observed data	Supplementary analysis was added	Section 7.1.2, 7.2.2
		To summarize TEAE leading to study drug discontinuation based on drug-related hepatic disorder.	A summary table was added	Section 8.1.1
		Pick up the latest version (2022) of Medical Queries from FDA	Webpage link was updated	Section 8.1.2
		To update modified Hy's Law criteria	Criteria were revised	Section 8.3
		To analyze liver function tests over time	Spaghetti plot was added	Section 8.3
		Protocol won't be amended	Section 10 was removed	Section 10
		To clarify classification algorithm of prohibited/restricted medications	Two classification tables were added	Appendix 3

1 Introduction

This statistical analysis plan (SAP) describes the statistical methods, assumptions, and details of the planned primary and final analyses to be conducted for the Study CEXV811A12201 [formerly CHK01-03 (ASSIST)]. Study CEXV811A12201 protocol amendment 02 (v03) is available at the time of finalization of this SAP. Hypothesis testing of the primary endpoint will be limited to the primary analysis.

2 Study Design

2.1 Description

This is a Phase 2, randomized, double-blind, placebo-controlled, crossover design to compare the efficacy and safety of atrasentan to placebo in subjects with IgAN on background therapy with SGLT2i. Adult male or female subjects 18 years or older with biopsy proven IgAN and estimated glomerular filtration rate (eGFR) of at least 30 mL/min/1.73 m^2 may be eligible for the study (see protocol for full eligibility criteria). All subjects participating in the study will be required to be on a maximally tolerated and stable dose of a renin-angiotensin system inhibitor (RASi) (physician choice) throughout the study. All subjects must have >0.5 grams/day total urine protein as measured on a 24-hour urine collection prior to enrollment in the trial.

Subjects eligible for this study may be on a stable dose of SGLT2i prior to screening, may have received SGLT2 inhibitors in the past but not be on a stable dose, or be SGLT2i naïve. Subjects who are not on or have not received stable doses of SGLT2i prior to study entry must have 24-hour total urine protein of >0.85 grams/day at screening prior to the run-in period and will enter the run-in period during which they will receive SGLT2i treatment for 8 weeks. The choice of SGLT2i will be at the discretion of the principal investigator and per local treatment guidelines.

Subjects who participate in the run-in period will be required to meet the criterion of a 24-hour total urine protein of >0.5 grams/day, which will be confirmed at the end of the run-in period. Additionally, the eGFR requirement of at least 30 mL/min/1.73 m^2 will be re-confirmed at the end of the run-in period.

All eligible subjects will be randomized 1:1 to either sequence AB or sequence BA in which they will receive 0.75 mg atrasentan once daily (QD) during one period and matching placebo during the other period as determined by the randomization schema. Randomization will be stratified by SGLT2i status (stable vs not stable). All subjects will enter Treatment Period 1 for 12 weeks. Thereafter, all subjects will enter a 12-week washout period before entering Treatment Period 2 for 24 weeks. Following the end of treatment, subjects will have follow-up evaluations for safety approximately 4 weeks after the end of treatment.

Individual subject participation duration: up to 66 weeks for subjects completing the SGLT2i run-in period and 58 weeks for subjects who do not participate in the run-in period

Treatment duration: 36 weeks of treatment with investigational product (IP)

The total duration for study participation per subject is expected to be up to 66 weeks for subjects completing the SGLT2i run-in period and 58 weeks for subjects who do not participate in the run-in period, and treatment duration with investigation product (IP) is expected to be 36 weeks:

Screening:	up to 6 weeks
SGLT2i Run-in Period[a]:	8 weeks
Treatment Period 1:	12 weeks
Washout Period:	12 weeks
Treatment Period 2:	24 weeks
End of study period:	4 weeks

[a] For subjects not on stable doses of SGLT2i

2.2 Treatment Assignment and Blinding

Eligible subjects will be randomly assigned in a 1:1 ratio to either sequence AB or sequence BA in which they will receive atrasentan 0.75 mg once daily (QD) during one period and matching placebo during the other period by the randomization schema. Randomization will be stratified by SGLT2i status (stable vs. run-in). A centralized Interactive Response Technology (IRT) system will be used to manage subject enrollment and randomization. Subjects and investigators will remain blinded to the initial treatment regimen for the entire duration of the study.

2.3 Study Objectives and Endpoints

Objectives	Endpoints
Primary	
• To evaluate the efficacy of atrasentan vs placebo while on background therapy with SGLT2i	• The change in proteinuria (UPCR from a 24-hour urine collection) from Baseline to Week 12
Secondary	
• To evaluate the efficacy of atrasentan vs placebo while on background therapy with SGLT2i at 24 weeks of treatment	• In Treatment Period 2, the change in proteinuria (UPCR from a 24-hour urine collection) from Baseline to Week 24

Objectives	Endpoints
Safety	
• To evaluate the safety of atrasentan vs placebo while on background therapy with SGLT2i	• Type, incidence, severity, seriousness, and relatedness of adverse events (AEs)
Pharmacokinetics	
• To evaluate the steady-state pharmacokinetics (PK) of atrasentan that support exploratory exposure-response analyses	• Atrasentan plasma levels
Exploratory	
• To evaluate the effect on eGFR between atrasentan vs placebo while on background therapy with SGLT2i at 24 weeks of treatment	• In Treatment Period 2, the change in eGFR of atrasentan vs placebo using the chronic kidney disease-epidemiology collaboration (CKD-EPI) creatinine equation from Baseline to Week 24
• To evaluate additional safety outcomes	• Incidence, severity, seriousness, and relatedness of adverse events of special interest including events of fluid overload<br>• Clinically significant changes in:<br>o Safety laboratory (hematology, complete chemistry and urinalysis) and BNP
Exploratory	
CCI [redacted]	

2.4 Sample Size

A target of approximately 52 subjects will be enrolled into the study to ensure a clinically meaningful difference can be detected between atrasentan and placebo in the primary endpoint of proteinuria reduction (UPCR) at Week 12 in addition to the secondary endpoint of 24-week UPCR change from baseline in study Treatment Period 2.

Approximately 52 subjects (approximately 26 per treatment sequence) will provide approximately 83% power using a two-sided pairwise test to detect a treatment effect of at least 0.288 in natural log transformed UPCR (25% reduction) from Baseline to Week 12 between atrasentan versus placebo at the 5% significance level, assuming a standard deviation of 0.67 and 5% rate of early withdrawal from the study before Week 12 in Treatment Period 2.

Using the same assumptions as described above and 5% rate of early withdrawal before Week 24 in Treatment Period 2, 52 subjects will also provide approximately 80% power using a two-sided two-sample t-test to detect a treatment effect of at least 0.56 in natural log transformed UPCR (43% reduction) over 24 weeks in study Treatment Period 2.

The assumptions used for sample size calculations are supported by the results observed in the previous Phase 3 and Phase 2 studies of atrasentan conducted in subjects with diabetic kidney disease (de Zeeuw, 2014; Heerspink, 2019; Lin, 2018) and in Phase 2 studies in IgAN.

2.5 Schedule of analyses

2.5.1 Final Analysis

The final, unblinded analysis will be performed after all subjects have completed (or discontinued) the study. The primary endpoint of the change from baseline in UPCR at Week 12 will be tested at a significance level of 0.05 (two-sided), The hypothesis testing of the key secondary endpoint, the change from baseline in UPCR at Week 24 will be conducted at a significance level of 0.05 (two-sided) without adjustment for multiplicity.

2.5.2 Multiple Comparisons and Type I Error Control

No multiplicity adjustments will be done for this Phase 2 study.

3 Definitions, Derivations, and Data-Handling Conventions

3.1 Analysis Sets

3.1.1 Screened Analysis Set

The Screened analyses set is defined as all subjects who provided written informed consent.

3.1.2 Intent-to-Treat Analysis Set

The Intent-to-Treat (ITT) analysis set is defined as all subjects randomly assigned to a treatment sequence. Subjects will be analyzed by the treatment sequence assigned at randomization.

3.1.3 Run-In Analysis Set

The Run-In analysis set is defined as all subjects who took at least one dose of SGLT2i in the run-in period. The Run-In analysis set will be used for the run-in period summaries.

3.1.4 Safety Analysis Set

The Safety analysis set is defined as all subjects randomly assigned to a treatment sequence who took at least one dose of study drug. For comparisons by treatment sequence, subjects will be analyzed according to the intervention they actually received, defined as the randomized treatment sequence if it was received at least once and otherwise defined as the actual treatment received for the duration of the study. For comparisons by treatment period, subjects will be analyzed according to the intervention they actually received.

3.1.5 Pharmacokinetic Analysis Set

The Pharmacokinetic (PK) analysis set is defined as all subjects in the Safety analysis set who have at least one non-missing concentration value reported by the PK laboratory. Subjects will be analyzed according to the intervention they received.

CCI [redacted]

3.2 Subgroups

There will be no subgroup analyses.

3.3 Study Date Related Definitions

3.3.1 Date of First and Last Dose of Treatment

Date of first dose of study treatment in each treatment period is defined as the first date when a dose of the study treatment (atrasentan or placebo) was administered. If the day and month of the first dose date is missing, the first dose date will be imputed to be the randomization date for Treatment Period 1 and date of last dose in Treatment Period 1 + 85 days for Treatment Period 2. If the day of the first dose date is missing in Treatment Period 1, the first dose date will be imputed to be the randomization date if the month of the first dose date is the same as the month of randomization and otherwise will be imputed to the first day of the month. If the day of the first dose is missing in Treatment Period 2, it will be imputed to be the date of Treatment Period 2 baseline if the month of the first dose date is the same as the month of Treatment Period 2 baseline and otherwise will be imputed to the first day of the month.

Date of last dose of study treatment in each treatment period is defined as the last date when a dose of the study treatment (atrasentan or placebo) was administered. The date of last dose in Treatment Period 1 and Treatment Period 2 will be determined from the End of Treatment (EOT) eCRF.

The date of the first dose of SGLT2i in the run-in period will be determined from the Concomitant Medications (SGLT2i Auxiliary Medications) eCRF.

3.3.2 Date of Last Contact

The date of last contact for the final analysis will be the date of study completion or discontinuation from the End of Study (EOS) eCRF.

3.3.3 Study Day

For each subject, the study day of a specific study assessment is defined as the number of days from the first dose date in Treatment Period 1.

For dates on or after the first dose date, study day will be calculated as:

Study day = Date of study assessment – first dose date +1.

For dates prior to the first dose date, study day will be represented as a negative number and will be calculated as:

Study day = Date of study assessment – first dose date.

There will be no Study Day 0.

3.3.4 Baseline

In general, 'baseline' is defined as the last non-missing value on or prior to the day of the first dose of study treatment in each treatment period unless otherwise specified. Note, the baseline value of treatment period 2 should not be derived from non-missing value during treatment period 1 or earlier, or within the first 30 days after the last dose of treatment period 1. The washout period will use the baseline of Treatment Period 1. Exceptions apply to the following parameters in the Treatment Period 1 and Treatment Period 2:

- **24-hour Urine - UPCR, Urine albumin:creatinine ratio (UACR), and Total Protein:** any assessment out of 28 days from Day 1 visit date won't be used for baseline derivation. When two samples are available on or prior to the day of the first dose of study in a treatment period, the baseline value for each of these lab parameters is defined as the geometric mean of the two 24-hour urine samples collected closest in time but prior to the first dose on Day 1 that were collected 28 days or less apart. If the two samples were collected greater than 28 days apart, then the test result for the sample collected closest in time but prior to the first dose on Day 1 will be used as the baseline. If only one sample was collected prior to the first dose on Day 1, then this test result will be used as the baseline value.
- **24-hour Urine - UPCR, UACR, and Total Protein– Natural Log-scale:** baseline value on the natural log-scale for each of the above lab parameters, is defined as the natural logarithm of the baseline value as defined above for the untransformed values.

- **FMV Urine - UPCR, UACR:** any assessment out of 28 days from Day 1 visit date won't be used for baseline derivation. Unscheduled visit samples won't be used unless there is no scheduled visit assessment on or prior to first dose. When two samples are available on or prior to the day of the first dose of study in a treatment period, the baseline value for each of these lab parameters is defined as the geometric mean of the two FMV urine samples collected closest in time but prior to the first dose on Day 1 that were collected 28 days or less apart. If the two samples were collected greater than 28 days apart, then the test result for the sample collected closest in time but prior to the first dose on Day 1 will be used as the baseline. If only one sample was collected prior to the first dose on Day 1, then this test result will be used as the baseline value.
- **FMV Urine - UPCR and UACR – Natural Log-scale:** baseline value on the natural log-scale is defined as the natural logarithm of the baseline value as defined above for the untransformed value of FMV urine collections.
- **Other 24-hour Urine Parameters:** any assessment out of 28 days from Day 1 visit date won't be used for baseline derivation. When two samples are available on or prior to the day of the first dose of study in a treatment period, the baseline value for each of the 24-hour urine lab parameters except for UPCR, UACR and Total Protein is defined as the arithmetic mean of the test results from the two 24-hour urine samples collected closest in time but prior to the first dose on Day 1 that were collected 28 days or less apart. If the two samples were collected greater than 28 days apart, then the test result for the sample collected closest in time but prior to the first dose on Day 1 will be used as the baseline. If only one sample was collected prior to the first dose on Day 1, then this test result will be used as the baseline value.
- **eGFR**: any assessment out of 28 days from Day 1 visit date won't be used for baseline derivation. Unscheduled visit samples won't be used unless there is no scheduled visit assessment on or prior to first dose. When two samples are available on or prior to the day of the first dose of study in a treatment period, baseline eGFR is defined as the mean of the eGFR results from the two assessments closest in time but prior to the first dose on Day 1 that were assessed 28 days or less apart. If these two assessments were collected greater than 28 days apart, then the eGFR result for the assessment closest in time but prior to the first dose on Day 1 will be used as the baseline. If only one assessment was collected prior to first dose on Day 1, then this eGFR result will be used as the baseline value.

3.3.5 Post-Baseline

- **Week 12 (Treatment Period 1 and Treatment Period 2) and Week 24 (Treatment Period 2) UPCR, UACR, and Total Protein (24-hour Urine):** the value for each of these lab parameters at their respective post-baseline timepoints is defined as the geometric mean of the 2 separate test results from 24-hour urine samples that were collected 10 days or less apart for these study visit assessments. If the two samples were collected greater than 10 days apart, then the test result from the first sample will be used as the analysis value. If one of the 24-hour urine samples is missing, the single test result will be used for the visit assessment.

- **Week 12 (Treatment Period 1 and Treatment Period 2) and Week 24 (Treatment Period 2) UPCR, UACR, and Total Protein (24-hour Urine) – Natural Log-Scale:** the value on the natural log-scale for each of the above lab parameters at their respective post-baseline timepoints is defined as the arithmetic mean of the natural logarithm of the values with identical timing considerations as defined above for the untransformed values.

- **Week 12 (Treatment Period 1 and Treatment Period 2) and Week 24 (Treatment Period 2) for Other 24-Hour Urine Parameters:** the value for each of the 24-hour urine lab parameters at their respective post-baseline timepoints except for UPCR, UACR and Total Protein is defined as the arithmetic mean of the 2 separate test results from 24-hour urine samples that were collected 10 days or less apart for these study visit assessments. If the two samples were collected greater than 10 days apart, then the test result from the first sample will be used as the analysis value. If one of the 24-hour urine samples is missing, the single test result will be used for the visit assessment.

Post-baseline summaries over the Treatment Period 1 or 2 (except by-visit summaries) will include the first 30 days after the last study treatment of Treatment Period 1 or 2. Post-baseline summaries over the Washout Period (except by-visit summaries) will exclude the first 30 days after the last study treatment of Treatment Period 1.

3.4 Data Handling Conventions

3.4.1 Missing Data

3.4.1.1 Missing Results

All efforts will be made to prevent missing data. Missing data for continuous safety assessments such as laboratory and vital signs data will not be imputed unless otherwise specified.

- **UPCR (24-hour urine):** In the event of completely missing 24-hour urine samples for baseline, the UPCR based on first morning void (FMV) from the last assessment prior to the first dose of study treatment will be used in their place, if available and as applicable in each treatment period. Any assessment out of 28 days from Day 1 visit date won't be used for baseline derivation.

3.4.1.2 Missing and Partial Dates

- **Dosing Dates:** The handling of missing and partial dose dates is described in Section 3.3.1.

- **Adverse Event, Medication, and Procedure Dates:** Missing and partial start and stop dates for AEs and medications will be imputed as described in Table 1 below.

Table 1: Start and End Date Imputation

Date	Missing Component(s)	Scenario	Imputation
Start date	Day	• *Start month* = month of 1st dose and<br>• *Start year* = year of 1st dose in either treatment period or run-in period	Impute *start day* as day of 1st dose in corresponding period (i.e., *start date* = date of 1st dose)
		• *Start month* ≠ month of 1st dose in either treatment period or run-in period	Impute *start day* as 1st day of the *start month*
	Day and month	• *Start year* = year of 1st Dose in either treatment period or run-in period	If *start year* = year of 1st dose in run-in period = year of 1st dose in treatment period 1, then impute *start date* as date of 1st dose in treatment period 1<br>Otherwise, Impute *start date* as date of 1st dose in the first corresponding period
		• *Start year* ≠ year of 1st Dose in either treatment period or run-in period	Impute *start day* and *month* as 01 January
	Day, month, and year	NA	Not imputed
End date	Day	NA	Set *end day* as the last day of the *end month* (e.g., 30, 31, etc.) and then select the earliest of this date, date of last dose of study treatment + 30 days, and the data cut-off date
	Day and month	NA	Set *end day* and *month* to 31 December and select the earliest of this date, date of last dose of study treatment + 30 days date, and the data cut-off date
	Day, month, and year and not marked as 'Ongoing'	NA	Date not imputed

In conjunction with the imputation rules in Table 1 above, the following conventions will be applied:

- If the imputed start date is after the non-imputed stop date, then the start date will be set equal to the stop date
- If the imputed end date is prior to the non-imputed start date, then the stop date will be set equal to the start date

- **Procedure Dates**
  - Start date imputation should follow the rules in Table 1
  - If only the start date is collected, then the end date will be set equal to the start date
  - If both the start date and the end date are collected, then imputation of the end date should follow the rules in Table 1
- **Diagnosis Dates**
  - If *diagnosis day* is missing, set *day* to "01"
  - If *diagnosis day* and *month* are missing the set *day* and *month* to "01 January"

3.4.2 Data Transformations and Conventions

Only central lab data will be used in the analyses of the primary and secondary efficacy endpoints. Laboratory data that is below the lower limit of quantification (LLOQ) or higher than the upper limit of quantification (ULOQ) will be imputed to one unit less than the LLOQ or one unit more than the ULOQ, respectively. For example, a value of <11 will be assigned to 10 and a value of <11.0 will be assigned as 10.9. An exception to this rule is any value reported as <1, or <0.1, etc. For values reported as < 1 or < 0.1, a value of 0.9 or 0.09, respectively, will be used to calculate summary statistics. The LLOQ value or ULOQ will be used to calculate descriptive statistics if the datum is reported in the form of "≤ x" or "≥ x" (where x is considered the LLOQ or ULOQ, respectively).

- **UPCR, UACR, and Total Protein (24-hour Urine):** For analysis purposes, UPCR, UACR, and total urine protein results will be transformed using the natural logarithm and the change from baseline calculated using the log-transformed values. Baseline will be determined as defined in Section 3.3.4.
- **eGFR Calculation:** The CKD-EPI Creatinine Equation (Inker, 2021) will be used by the central laboratory to calculate eGFR (mL/min/1.73 m^2). The equation is as follows:

$$eGFR = 142 \times \min\left(\frac{S_{cr}}{\kappa}, 1\right)^{\alpha} \times \max\left(\frac{S_{cr}}{\kappa}, 1\right)^{-1.200} \times 0.9938^{Age} \times 1.012[if\ female]$$

  Where:

  - S_{cr} = standardized serum creatinine (mg/dL)
  - κ = 0.7 (females) or 0.9 (males)
  - α = -0.241 (females) or -0.302 (males)
  - min() indicates the minimum

- max() indicates the maximum
- age in years

- **Geometric mean (GM)**: The geometric mean is calculated as follows:

  $GM = [y_1 \times y_2 \times \ldots \times y_n]^{1/n} = \exp[\ln(GM)]$

  where $\ln(GM) = 1/n\ [\ln(y_1) + \ln(y_2) + \ldots + \ln(y_n)]$ and ln = natural logarithm

- **Geometric Mean Coefficient of Variation (GM CV):**

  $GM\ CV = (\exp[(\text{standard deviation})^2]-1)^{1/2}$

3.4.3 Classification of prohibited/restricted medications

Systemic steroids, other immunosuppressive therapy, and investigational or approved products for the treatment of IgAN will be identified using the World Health Organization (WHO) Drug Dictionary (Global B3 March 2021 version or higher). The specific ATC codes, preferred terms, and duration criteria that will be used to programmatically identify and determine whether a prohibited/restricted medication meets the criteria for the intercurrent event, Initiation of prohibited or restricted concomitant medication, are described in Appendix 3.

3.5 Analysis Conventions

3.5.1 Study Visits

The nominal visit as recorded on the eCRF will be used when data are summarized by visit. For central laboratory results, the nominal visit recorded in the laboratory dataset will be used. Any data relating to unscheduled visits will not be assigned to a particular visit or time point. However, the following exceptions will be made:

- An unscheduled visit prior to the first dose of study drug may be included in the calculation of the baseline value in each treatment period, if applicable.
- For subjects who prematurely discontinue from the study, early termination laboratory and vital signs data will be assigned to the applicable visit window in Table 2 below.

If multiple valid, non-missing measurements exist for a visit, records will be chosen based on the following rules if a single value is needed:

- For baseline, the last non-missing value on or prior to the first dose date of study drug in each treatment period will be selected, unless specified differently. If there are multiple records with the same time or no time recorded on the same day, the baseline value will be the average of the measurements for continuous data, or the measurement with the lowest severity (e.g. normal category (negative) will be selected over abnormal categories (large, moderate, small) for occult blood) for categorical data.
- For post-baseline values:
  - The record closest to the nominal day for that visit will be selected.
  - If there are two records that are equidistant from the nominal day (and both are records reported while on treatment), the later record will be selected.

- If there is more than one record on the selected day with the same time or no time recorded on the same day, the average will be taken for continuous data and the worse severity (e.g. within abnormal categories, large will be selected over moderate for occult blood) will be taken for categorical data, unless otherwise specified.

- For UPCR, eGFR, laboratory, and vital sign results collected at end of treatment (EOT) or end of study (EOS) visits for subjects who prematurely discontinue treatment, the following visit windows will be used:

Table 2: Visit Windows

Scheduled Assessment	Window	
	Weeks	Days
Run-In, Week 4	1 – 6	7 – 42
Run-In, Week 8*	>6	>42
Treatment Period 1, Week 2	1 – 4	7 – 28
Treatment Period 1, Week 6	>4 – 9	>28 – 63
Treatment Period 1, Week 12*	>9	>63
Washout Period, Week 6*	>1	>7
Treatment Period 2, Week 2	1 – 4	7 – 28
Treatment Period 2, Week 6	>4 – 9	>28 – 63
Treatment Period 2, Week 12	>9 – 18	>63 – 126
Treatment Period 2, Week 24	>18 – 26	>126 – 182
Follow-up, Week 28	>26	>182

* Upper bound of the window is the last day of the respective study period.

If multiple assessments fall within the same visit window, then the assessment closest in distance to the intended week of that scheduled assessment will be assigned to the visit.

3.5.2 General Statistical Methods

3.5.2.1 Continuous Data

Continuous data (e.g., age, weight, height) will be summarized using the following descriptive statistics among non-missing data: sample size, mean, standard deviation, median, 1st quartile (Q1), 3rd quartile (Q3), minimum, maximum.

Only one raw or mean observation per subject per time point will be used to compute these summaries. These summaries will be referred to as continuous summaries.

3.5.2.2 Categorical Data

Categorical data (e.g., gender, race, AEs) will be summarized using frequencies and percentages. Each subject will be counted only once in any category, for all variables, unless otherwise specified. These summaries will be referred to as categorical summaries.

3.5.2.3 Longitudinal Repeated Measurements

Efficacy longitudinal repeated measurements will be analyzed using a mixed-effects model repeated-measures (MMRM) model, unless otherwise specified. In SAS®, the PROC MIXED procedure will be used for the MMRM analysis including changes from baseline at each scheduled post-baseline timepoint within each treatment period. The covariance structure will be assumed to be unstructured (TYPE=UN) to estimate the within subject correlations. In cases where models do not converge, the following covariance structures will be tested: first-order autoregressive with heterogenous variances (TYPE = ARH(1)) followed by first-order autoregressive with homogenous variances (TYPE = AR(1)). The Kenward-Rogers method for computing the denominator degrees of freedom for the test of fixed effects will be used, and the Type III sums-of-squares for the Least Squares means (LSMeans) will be used to estimate treatment group differences. This approach will be referred to as MMRM.

3.6 Other General Definitions

3.6.1 Run-in period

Run-in period begins at the first dose of SGLT2i and ends with (randomization date -1).

3.6.2 Treatment period 1

Treatment Period 1 begins at the time of randomization and ends with Treatment Period 1 Week 12/EOT.

3.6.3 Washout period

Washout period begins at (Treatment Period 1 Week 12/EOT + 1 day) and ends with (Treatment Period 2 Day 1 - 1 day).

3.6.4 Treatment period 2

Treatment Period 2 begins at Treatment Period 2 Day 1 and end with Treatment Period 2 Week 24/EOT.

3.6.5 Safety follow-up period

Safety follow-up period begins at (Treatment period 2 Week 24/EOT + 1) and ends with Week 28/EOS.

3.6.6 On-treatment values

On-treatment values are defined as the results collected in the interval between the first dose and the last dose of study drug + 7 days for each treatment period.

3.6.7 Last post-treatment values

For laboratory data analysis, last post-treatment values are defined as the last result collected in the interval between 8 and 30 days after last dose of study drug for each treatment period.

3.6.8 Data for safety reporting

For safety analysis, treatment period data begins at 1st dose date and ends at last dose + 30 days for each treatment period.

For safety analysis, washout period data begins at 31st days after last dose of study treatment in Treatment Period 1, and ends prior to 1st dose of Treatment Period 2.

4 Subject Disposition and Enrollment

The following information will be summarized by treatment sequence. The summary will be based on the ITT analysis set unless otherwise specified.

4.1 Disposition

The following information will be summarized by treatment sequence, as applicable:

- Number of subjects screened
- Number of SGLT2i stable screened subjects

  Number of SGLT2i stable screened subjects subsequently randomized

  Number of SGLT2i stable screened subjects not randomized
- Number of SGLT2i run-in screened subjects
  - Number of SGLT2i run-in subjects who received at least one dose of SGLT2i in the run-in period
    - Number of run-in complete and subsequently randomized subjects
    - Number of run-in failure subjects
  - Number of SGLT2i run-in failure subjects
- Number of subjects randomized (by treatment sequence)
- Number and percentage of subjects in each analysis set
- Number and percentage of subjects who completed the Treatment Period 1, Washout period and Treatment Period 2
- Number and percentage of subjects who completed the study
- Number and percentage of subjects who prematurely discontinued the study and the associated reasons for discontinuation

In addition, the reasons for screen failures will be provided.

The following information will be summarized by treatment sequence and study period:

- Number and percentage of subjects who initiated the study treatment
- Number and percentage of subjects who completed study treatment
- Number and percentage of subjects who prematurely discontinued study treatment and the associated reasons for discontinuation

Listings of subjects who prematurely discontinued study treatment and/or the study will be provided.

4.2 Enrollment and Randomization

The following information will be summarized by treatment sequence:

- Number and percentage of subjects randomized by region (Asia, North America, Latin America, Europe, Oceania), country (within a region), and investigator/institution (within a region and county)
- Number and percentage of subjects in each stratum of the randomization stratification variable SGLT2i status (stable vs. run-in)

4.3 Protocol Deviations

The number and percentage of subjects with at least one important protocol deviation and the number and percentage of subjects within each protocol deviation category will be summarized by treatment sequence.

A listing will be provided of subjects who had at least one important protocol deviation. A listing of protocol deviations due to COVID-19 will be provided.

4.4 Medical History

General medical history and IgAN history will be collected at screening. Medical history will be coded using the Medical Dictionary for Regulatory Activities (MedDRA), Version 24.0 or higher. Medical history will be summarized by MedDRA system organ class (SOC) and within SOC by descending frequency of MedDRA preferred term for each treatment sequence and overall. Listings of IgAN history will be provided.

5 Demographics and Baseline Characteristics

Demographic and baseline characteristics information will be summarized based on the ITT analysis set unless otherwise specified.

The following demographics will be summarized by treatment sequence:

- Age
- Age group (<65, ≥65)

- Gender
- Race
- Ethnicity
- Geographic region (Asia, Europe, Northern America, Latin America and the Caribbean, Oceania)

The following baseline characteristics will be summarized by treatment sequence and study period:

- Weight
- Body Mass Index (BMI)
- Systolic blood pressure (SBP)
- Systolic blood pressure category (≤ 110, > 110-<140, ≥ 140)
- Diastolic blood pressure (DBP)
- Diastolic blood pressure category (<70, 70-<80, 80-<90, ≥ 90)
- Blood pressure category (SBP ≥ 140 or DBP ≥ 90; SBP <140 and DBP <90)
- Laboratory assessments (e.g., 24-hour UPCR (g/g), 24-hour total urine protein (mg/day), 24-hour UACR (g/g), FMV UACR (g/g), FMV UPCR (g/g), eGFR (mL/min/1.73 m^2), brain natriuretic peptide (BNP) (ng/L), hemoglobin (g/dL)), FMV urinary red blood cells, FMV urinary blood.
- Baseline 24-hour UPCR category (<, >= 1.5 g/g) and (< 1 g/g, 1 -< 2 g/g, 2-<3 g/g, >= 3 g/g)
- FMV UPCR category (< 1 g/g, 1-<2 g/g, 2-<3 g/g, >= 3 g/g)
- 24-hour total urine protein category (< 1 g/day, 1-<2 g/day, 2 -<3 g/day, >= 3 g/day)
- Baseline eGFR category (≤ 45 mL/min/1.73 m^2, > 45-≤ 60 mL/min/1.73m^2, > 60 mL/min/1.73m^2)
- RAS inhibitor usage (no RAS inhibitor usage vs. angiotensin-converting enzyme (ACE) inhibitor usage only vs. angiotensin II receptor blocker (ARB) usage only vs. direct renin inhibitor usage)
- Baseline medication of special interest (SGLT2i, RAS Inhibitor, Diuretics, Immunosuppressants including systemic corticosteroids, Blood pressure lowering agents)

The following baseline characteristics will be summarized by treatment sequence for Treatment Period 1 only:

- Height

- Duration of disease
- glycated hemoglobin (HbA1c) (%)
- ECG
- MEST-C score

Continuous data will be summarized as described in Section 3.5.2.1 and categorical data will be summarized as described in Section 3.5.2.2. Listings of demographic and baseline characteristic will be provided.

The duration of disease, defined as the time from the most recent biopsy (first dose date in Treatment Period 1 – most recent biopsy date)/365.25, will be summarized by treatment sequence. For subjects that were never dosed, the randomization date will be used instead of the first dose date.

6 Study Treatment Exposure and Compliance

The following study treatment related information will be calculated for each subject and summarized by treatment sequence and study period based on the Safety analysis set unless otherwise specified.

- On-treatment compliance (%) = (Total # of tablets taken ÷ Total # of tablets expected to be taken while on treatment) x 100
- Uncertainty (%) = (number of bottles with no site reconciliation date)/(total number of bottles in treatment period dispensed) x 100
- Duration of exposure = date of last dose – date of first dose +1

In calculation of duration of exposure, the first and the last dose dates will follow definitions in Section 3.3.1. Participants with % uncertainty in terms of drug accountability (≥ 30%) are excluded from the compliance calculation. Total # of tablets taken refers to quantity dosed. The expected number of tablets taken while on treatment will be calculated as duration of exposure x 1 tablet QD. The total amount of study drug administered, duration of exposure, and on-treatment compliance will be summarized using continuous summaries as described in Section 3.5.2.1. In addition, on-treatment compliance will be summarized by category (e.g., < 70%, 70%- <80%, 80% – <90 %, ≥ 90%) with the number and percentage of subjects in each category provided. A cumulative summary of the number and percentage of subjects in each of the following duration of exposure categories will also be provided by treatment sequence (e.g., ≥1 day, ≥4 weeks, ≥8 weeks, ≥12 weeks, ≥24 weeks).

7 Analysis of Efficacy

Efficacy analyses will be by treatment group and based on the ITT analysis set unless otherwise specified.

7.1 Primary Endpoint

The primary efficacy endpoint is the change in proteinuria (UPCR based on 24-hour urine collection) from Baseline to Week 12 across both periods between atrasentan versus placebo. The formal, primary analysis of this endpoint will be conducted as described in Section 2.5. The hypothesis test will be based on a 2-sided significance level of 0.05.

Subjects missing their Baseline UPCR or with no post-Baseline UPCR results will be excluded from the analyses of this endpoint unless otherwise specified. All UPCR results at Baseline and scheduled Week 12 visits in each study period will be included in the primary analysis.

Subjects in the AB sequence (atrasentan-placebo) receive atrasentan in treatment period 1, and after the effects of atrasentan have subsided, they receive placebo in treatment period 2. The remaining subjects assigned to the BA sequence (placebo-atrasentan) receive treatment Placebo first and then atrasentan. Therefore, the difference in atrasentan and placebo is derived from a within-subject comparison.

The final analysis of this primary efficacy endpoint will be performed using a mixed model. The mixed model will include the change from baseline of natural log UPCR at Week 12 within each treatment period as an outcome. Baseline and Week 12 natural log UPCR will be calculated as described in Section 3.3.4 and Section 3.3.5, respectively. The model will also include the fixed effects of sequence, period, and treatment, a random effect of subjects nested within sequences, and covariates of Baseline natural log UPCR as a continuous variable and the randomization stratification factor of SGLT2i status (stable vs run-in). The covariance structure is not applicable because only one post-baseline measurement (Week 12) is analyzed over each treatment period.

From this model, least squares means (LSMeans), standard errors, treatment differences in LSMeans, and 95% confidence intervals will be estimated and displayed. LSMeans for change from baseline on the natural log scale and associated 95% confidence intervals will be transformed into percent change from baseline as 100*(exp(LSMeans) – 1). Estimated within-subject treatment differences (atrasentan vs placebo) along with corresponding 2-sided 95% CIs and p-values will also be presented and will be estimated as the difference in LSMeans between atrasentan and placebo. The within-subject difference in percent change in UPCR between atrasentan and placebo will be estimated by 100*[exp(diff) -1] where 'diff' is the treatment difference as estimated by the difference in LSMeans between atrasentan and placebo. The between group (atrasentan vs. placebo) geometric mean change in 24-hour UPCR based on within-subject difference will be derived as 100*(exp[least square mean change]-1), and the same transformation will be applied to the 95% confidence limits.

To evaluate period effect, by each treatment sequence, UPCR in a logarithmic scale change from baseline (within each treatment period) will be analyzed based on a mixed model using treatment as fixed-effect factors, baseline natural log UPCR and the randomization stratification factor of SGLT2i status (stable vs run-in) as covariates, with a random effect of subjects.

Rubin's rules will be applied to the estimates, measured by the ratio and 95% CI of Week 12 vs. baseline and a within-individual ratio and 95% CI of atrasentan vs. placebo.

Observed 24hr UPCR will be summarized and plotted by treatment period. It will also be summarized by treatment group. In addition, a period effect table will be summarized by treatment sequence and treatment period, measured by the ratio and 95% CI of Week 12 vs. baseline and atrasentan vs. placebo.

Primary inference will be based on the treatment comparison of the LSMeans at Week 12 from this model. The null hypothesis is that the mean difference in UPCR between the two treatment groups is zero, versus the alternative hypothesis that this difference is not equal to zero. The hypotheses can be expressed as follows:

H_0: $\mu_{atrasentan} - \mu_{placebo} = 0$

H_a: $\mu_{atrasentan} - \mu_{placebo} \neq 0$

7.1.1 Primary estimand

The estimand is the precise description of the treatment effect and reflects strategies to address events occurring during trial conduct which could impact the interpretation of the trial results (e.g., premature discontinuation of study drug).

Clinical Question: What will be the expected effect of being assigned atrasentan, compared to placebo, on UPCR reduction at Week 12 for the target population in the absence of investigational or approved products for the treatment of IgAN?

Justification: Treatment effect assessed in real-world clinical practice including the effects of study drug discontinuation, initiation of maintenance dialysis or kidney transplantation, dose modification or discontinuation of background therapies, and initiation of prohibited or restricted concomitant medications in conditions similar to clinical practice, but in absence of any potential confounding effects of investigational or approved products for the treatment of IgAN.

The primary estimand for the primary endpoint and the handling of intercurrent events are defined below:

- **Population:** Adults with biopsy-proven IgAN at risk of progressive loss of renal function who are receiving background therapy with SGLT2i and RASi.
- **Endpoint:** Change in proteinuria (UPCR from a 24-hour urine collection) from Baseline to Week 12 (across both periods).
- **Treatment of interest:** The randomized treatment of 0.75 mg atrasentan once daily (QD) or placebo in addition to background therapy with SGLT2i and RASi.
- **Handling of intercurrent events**
    1. Study drug discontinuation for any reason: treatment policy strategy
    2. Initiation of maintenance dialysis or kidney transplantation: treatment policy strategy

3. Dose modification or discontinuation background therapy (RAS/SGLT2 inhibitors): treatment policy strategy

4. Initiation of prohibited or restricted concomitant medication: treatment policy strategy

5. Initiation of an investigational or approved product for the treatment of IgAN (other than RASi and SGLT2i): hypothetical strategy

- **Summary measure:** Ratio of geometric means relative to baseline between treatment groups. The summary measure will be converted to a percentage change as described in Section 7.1.

A listing of selected efficacy information will be produced; this may include (but is not limited to) the observed 24-hr UPCR measurements from baseline onwards and for the participants with an I/C, whether they took place post the first I/C or the first I/C handled with a hypothetical strategy.

Handling of missing values due to intercurrent events

In each treatment period, the following intercurrent events will be handled using treatment policy strategy: study drug discontinuation, initiation of maintenance dialysis or kidney transplantation, dose modification or discontinuation of background therapy (RAS/SGLT2 inhibitors), and initiation of a prohibited or restricted concomitant medication (excluding investigational or approved products for the treatment of IgAN). All data collected after these intercurrent events will be included in the analysis.

In each treatment period, the intercurrent event of initiation of an investigational or approved product for the treatment of IgAN will be handled using hypothetical strategy, and data collected following the onset of this intercurrent event will be excluded from analysis (set to "missing").

The "jump-to-reference" ("J2R") imputation approach ([Carpenter et al, 2013][Kenward, 2015]) will be used to impute values after the occurrence of this intercurrent event for subjects in the atrasentan arm. See Section 7.1.2 for details on the jump-to-reference imputation approach. Values for the placebo group will be handled under the missing-at-random (MAR) assumption. Missing data due to intercurrent events (e.g., study treatment discontinuation, initiation of prohibited or restricted medications) will be imputed to indicate a worsening in condition (i.e., the "jump-to-reference" ("J2R") imputation approach for subjects in the atrasentan arm and under MAR for the placebo group). For implementation, missing data following the onset of an intercurrent event will be considered as missing due to the intercurrent event, representing a conservative approach, since it cannot be excluded that missing values would have been reflecting worsening of the condition. Missing data not due to intercurrent events (e.g., administrative reasons such as unavailability of a sample or a missed visit) will be handled using missing at random for both treatment arms.

If the subject has multiple intercurrent events during a treatment period, missing data will be handled based on the first intercurrent event. Treatment policy strategy will be utilized when two intercurrents events with different strategies occur first and happen on the same date.

Intercurrent events will be summarized and listed by treatment period.

Handling of missing values not related to intercurrent events

Missing data due to any non-administrative reason (e.g., study discontinuation, subject refused, physician decision, other) will be imputed to indicate a worsening in condition (i.e., the “jump-to-reference” (“J2R”) imputation approach for subjects in atrasentan and under MAR for the placebo group). Missing data due to administrative reasons (e.g., missing data due to unavailability of a sample or a missed visit, technical collection issue/device or procedure, Outside Testing Stability, QNS - Quantity Not Sufficient| Unable to Calculate): data will be handled using missing at random.

7.1.2 Sensitivity and Supportive Analyses

Supplementary analyses of the primary endpoint may be conducted to assess the robustness of the results of the primary analysis, and the impact of intercurrent events. Two supplementary analysis are planned:

- Supplementary analysis 1: a treatment policy strategy will be used to handle all intercurrent events (including investigational or approved products for the treatment of IgAN). All data collected after the occurrence of intercurrent events will be included in the analysis. The “jump-to-reference” (“J2R”) imputation approach will be used to impute missing values after the occurrence of these intercurrent events for subjects in the atrasentan arm.
- Supplementary analysis 2: a hypothetical strategy will be used to handle the intercurrent events of maintenance dialysis or kidney transplantation or prohibited/restricted medication use, or use of an investigational or approved product for the treatment of IgAN, to assess the treatment effect of atrasentan, compared to placebo, in absence of these intercurrent events. Values after the above intercurrent events for treatment of atrasentan and placebo arm of IgAN will be excluded from analysis (set to “missing”). The “jump-to-reference” (“J2R”) imputation approach will be used to impute values after the occurrence of these intercurrent events for subjects in the atrasentan arm. Under this approach, values after the intake of such medications in the atrasentan group will be imputed assuming that a subject’s mean profile follows that of placebo subjects that remain in the study (i.e. the placebo group is used as the “reference” group).

In more detail, values following these intercurrent events are imputed for atrasentan subjects assuming the joint distribution of their outcome data, before and after the intercurrent, is multivariate normal (MVN) with mean vector from the atrasentan group up to the occurrence of the intercurrent event, and a mean vector following that observed for the reference group after the intercurrent event. The covariance matrix matches that of the atrasentan group for values prior to the intercurrent event and that of the “reference group” for the conditional components of the values after the intercurrent event, given the values before the intercurrent

event. Values for the placebo group will be handled under the missing-at-random (MAR) assumption.

Imputation will be done for each treatment period separately. By each treatment period, the imputation model will include treatment, visit, and treatment-by-visit interaction, and the randomization stratification factor of SGLT2i status (stable vs run-in).

Many imputed datasets will be created, with their number chosen based on computational feasibility (but at least 200). Each imputed dataset will be analyzed using the analysis model and the results will be combined using Rubin's rules.

The target population, the primary endpoint, the treatment of interest, intercurrent events other than maintenance dialysis or kidney transplantation, or prohibited/restricted medication use and the summary measure of the supplementary estimand are the same as for the primary estimand.

Supplementary analysis 3 is planned on observed datasets only. Data after the intercurrent event, initiation of an investigational or approved product for the treatment of IgAN (other than RASi and SGLT2i), will be handled by hypothetical strategy. The mixed model under MAR assumption will include the fixed effects of sequence, period, and treatment, a random effect of subjects nested within sequences, and covariates of Baseline natural log UPCR as a continuous variable and the randomization stratification factor of SGLT2i status (stable vs run-in). The covariance structure is assumed to be unstructured and the same in each treatment group.

Table 3: Handling Strategy for Intercurrent Events

#	Intercurrent events	Analysis			
		Main analysis	Supplementary analysis 1	Supplementary analysis 2	Supplementary analysis 3
1	Study drug discontinuation for any reason	treatment policy strategy	*	*	*
2	Initiation of maintenance dialysis or kidney transplantation	treatment policy strategy	*	hypothetical strategy	*
3	Dose modification or discontinuation background therapy (RAS/SGLT2 inhibitors)	treatment policy strategy	*	*	*
4	Initiation of prohibited or restricted concomitant medication	treatment policy strategy	*	hypothetical strategy	*
5	Initiation of an investigational or approved product for the treatment of IgAN (other than RASi and SGLT2i)	hypothetical strategy	treatment policy strategy	*	*

A star (*) in cells for supplementary analyses indicates that this cell entry is identical to the one for the main analysis (primary endpoint analysis).

7.2 Secondary Endpoint

The secondary efficacy endpoint is the change in proteinuria (UPCR based on 24-hour urine collection) from Baseline to Week 24 in Treatment Period 2 between atrasentan versus placebo. The formal analysis of this endpoint will be conducted at the final analysis as described in Section 2.5.2. The hypothesis test will be based on a 2-sided significance level of 0.05 without adjustment for multiplicity.

Subjects missing their Treatment Period 2 Baseline UPCR or with no post-Baseline UPCR results will be excluded from the analyses of this endpoint unless otherwise specified.

The analysis of this endpoint will be performed using an MMRM model. The MMRM model will include the change from Treatment Period 2 Baseline of natural log UPCR at each post-Baseline measurement through Week 24 in Treatment Period 2 as outcomes. Treatment Period 2 Baseline and post-Baseline natural log UPCR will be calculated as described in Section 3.3.4 and Section 3.3.5, respectively.

The model will also include the fixed effects of treatment, visit, and treatment-by-visit interaction, with covariates of Treatment Period 2 Baseline natural log UPCR and the randomization stratification factor of SGLT2i status (stable vs run-in). The covariance structure

is assumed to be unstructured and the same in each treatment group. In cases where models do not converge, the following covariance structures will be tested: first-order autoregressive with heterogenous variances (TYPE = ARH(1)) followed by first-order autoregressive with homogenous variances (TYPE = AR(1)).

From this model, LSMeans, standard errors, treatment differences in LSMeans, and 95% confidence intervals will be estimated and displayed for each visit. LSMeans for change from baseline on the natural log scale and associated 95% confidence intervals will be transformed into percent change from baseline as 100*(exp(LSMeans) – 1). Estimated treatment differences (atrasentan vs placebo) along with corresponding 2-sided 95% CIs and p-values will also be presented at each post-baseline visit and will be estimated as the difference in LSMeans between atrasentan and placebo. The percent change in UPCR between atrasentan and placebo will be estimated by 100*[exp(diff) -1] where 'diff' is the treatment difference as estimated by the difference in LSMeans between atrasentan and placebo. The between group geometric mean change in 24-hour UPCR will be derived as 100*(exp[least square mean change]-1), and the same transformation will be applied to the 95% confidence limits. Percent change from baseline will be plotted for treatment period 2.

Primary inference will be based on the treatment comparison of the LSMeans at Week 24 in Treatment Period 2 from this model. The null hypothesis is that the mean difference in UPCR between the two treatment groups is zero, versus the alternative hypothesis that this difference is not equal to zero. The hypotheses can be expressed as follows:

H_0: $\mu_{atrasentan} - \mu_{placebo} = 0$

H_a: $\mu_{atrasentan} - \mu_{placebo} \neq 0$

7.2.1 Secondary estimand

Clinical Question: What will be the expected effect of being assigned atrasentan, compared to placebo, on UPCR reduction for the target population regardless of study drug discontinuation for any reason, initiation of maintenance dialysis or kidney transplantation, dose modification or discontinuation of RAS inhibitor therapy or SGLT2i, or initiation of prohibited or restricted concomitant medications?

Justification: Treatment effect assessed in real-world clinical practice including the effects of study drug discontinuation, initiation of maintenance dialysis or kidney transplantation, dose modification or discontinuation of RAS inhibitor therapy or SGLT2i, initiation of prohibited or restricted concomitant medications in conditions similar to clinical practice.

The secondary estimand for the secondary endpoint and the handling of intercurrent events are defined below:

- **Population:** Adults with biopsy-proven IgAN at risk of progressive loss of renal function who are receiving background therapy with SGLT2i and RASi.
- **Endpoint:** Change in proteinuria (UPCR from a 24-hour urine collection) from Baseline to Week 24 of Treatment Period 2.
- **Treatment of interest:** The randomized treatment of 0.75 mg atrasentan once daily (QD) or placebo in addition to background therapy with SGLT2i and RASi.

- **Handling of intercurrent events**

  1. Study drug discontinuation for any reason: treatment policy strategy

  2. Initiation of maintenance dialysis or kidney transplantation: treatment policy strategy

  3. Dose modification or discontinuation background therapy (RAS/SGLT2 inhibitors): treatment policy strategy

  4. Initiation of prohibited or restricted concomitant medication: treatment policy strategy

  5. Initiation of an investigational or approved product for the treatment of IgAN (other than RASi and SGLT2i): hypothetical strategy

- **Summary measure:** Ratio of geometric means relative to baseline between treatment groups. The summary measure will be converted to a percentage change as described in Section 7.1.

Handling of missing values

The same strategies for the primary endpoint will be employed for handling missing data related to and not related to intercurrent events for the secondary endpoint.

7.2.2 Sensitivity and Supportive Analyses

A supportive analysis of the secondary efficacy endpoint will be conducted to assess the robustness of the results of the key secondary endpoint analysis by analyzing change from Baseline to Week 24 in Treatment Period 2. This sensitivity analysis will be conducted using analysis of covariance (ANCOVA) methodology on the outcome of change from Treatment Period 2 Baseline to Week 24 in natural log UPCR. The model will include the fixed effects of factors of Treatment Period 2 Baseline natural log UPCR, treatment and SGLT2i status (stable vs. run-in). The robust sandwich error estimator will be used to estimate standard errors utilizing the EMPIRICAL option in SAS® PROC MIXED.

A supplementary analysis is planned on observed datasets only. Data after the intercurrent event, initiation of an investigational or approved product for the treatment of IgAN (other than RASi and SGLT2i), will be handled by hypothetical strategy. The mixed model for repeated measures (MMRM) under MAR assumption will include the fixed effects of treatment, visit, treatment-by-visit interaction and covariates of Baseline natural log UPCR as a continuous variable and the randomization stratification factor of SGLT2i status (stable vs run-in). The covariance structure is assumed to be unstructured and the same in each treatment group.

7.3 Exploratory Endpoints

7.3.1 eGFR in Treatment Period 2

An exploratory efficacy endpoint is the change in eGFR of atrasentan vs placebo from Baseline to Week 24 In Treatment Period 2. eGFR will be calculated based on serum creatinine using the CKD-EPI equation (2021) (Section 3.4.2). The analysis of this endpoint will be conducted at the final analysis as described in Section 2.5.1.

Subjects missing their Treatment Period 2 Baseline eGFR result will be excluded from the analyses of this endpoint unless otherwise specified. All eGFR results from scheduled visits will be included in the eGFR analyses unless otherwise specified. Observed change from baseline will be summarized and plotted for Treatment Period 2.

The analysis of this endpoint will be performed using an MMRM model. Data after the intercurrent event, initiation of an investigational or approved product for the treatment of IgAN (other than RASi and SGLT2i), will be handled by hypothetical strategy. The MMRM model will include eGFR change from Treatment Period 2 Baseline at each post-Baseline measurement through Week 24 in Treatment Period 2 as outcomes. Treatment Period 2 Baseline and post-Baseline eGFR will be calculated as described in Section 3.3.4 and Section 3.3.5, respectively.

The model will also include the fixed effects of treatment, visit, and treatment-by-visit interaction, with covariates of Baseline natural log UPCR and Baseline eGFR as continuous variables and the randomization stratification factor of SGLT2i status (stable vs run-in). The covariance structure is assumed to be unstructured and the same in each treatment group. In cases where models do not converge, the following covariance structures will be tested: first-order autoregressive with heterogenous variances (TYPE = ARH(1)) followed by first-order autoregressive with homogenous variances (TYPE = AR(1)).

From this model, LSMeans, standard errors, treatment differences in LSMeans, and 95% confidence intervals will be estimated and displayed for each visit. Estimated treatment differences (atrasentan vs placebo) along with corresponding 2-sided 95% CIs and p-values will also be presented at each post-baseline visit and will be estimated as the difference in LSMeans between atrasentan and placebo. The estimand for this exploratory analysis will be the same as the secondary estimand with change in eGFR from Baseline to Week 24 of Treatment Period 2 as the endpoint and the difference in the mean eGFR change from Baseline to Week 24 of Treatment Period 2 between subjects randomized to atrasentan vs placebo as the summary measure.

CCI [redacted]

8 Safety

Safety will be summarized by treatment within each period based on the safety analysis set unless otherwise specified. If a patient received no treatment in one particular treatment period, the patient will not be included in the safety analyses for that treatment period. Additionally, if a patient received no treatment in Treatment Period 1, the patient will not be included in the safety analyses for washout period. Percentages will be calculated out of the number of patients treated in Treatment Period 1 and Treatment Period 2, respectively. Additionally, Run-In analysis set will be used for all run-in period summaries.

8.1 Adverse Events

Per the study protocol, all AEs (non-serious and serious) reported from after informed consent has been obtained until End of Study (EOS) visit will be collected. In addition, adverse events (AEs) or serious adverse events (SAEs) caused by a protocol-mandated intervention (e.g., procedures such as blood draws, discontinuation of medications) will be collected from the time the subject signed the study-specific informed consent.

All AEs and SAEs will be coded using the Medical Dictionary for Regulatory Activities (MedDRA), Version 24.0 or higher and summarized by: 1) by descending overall frequency of MedDRA preferred term (PT), and 2) alphabetically by MedDRA system organ class (SOC) and within SOC by descending frequency of MedDRA PT.

8.1.1 Treatment-Emergent AEs and Deaths

Treatment-Emergent adverse events (TEAEs) are defined as adverse events that first occur or worsen on or after the first dose of IP in each respective randomized treatment period and within 30 days after the last dose of IP administration in each treatment period. In addition, adverse events with completely missing start dates will be considered treatment-emergent as follows:

Table 4: Classification of treatment-emergent AEs

AE end date	Classification
Completely missing	Will not be considered as TEAEs for either Treatment Period 1 or 2; such events will be included in a stand-alone listing
On or later than the first dose of IP in Treatment Period 1 but earlier than the first dose of IP in Treatment Period 2	TEAE over Treatment Period 1
On or later than the first dose of IP in Treatment Period 2	TEAE over Treatment Period 2

Partial start and end dates will be imputed as described in Section 3.4.1.2.

The number and percentage of TEAEs will be summarized, with multiple occurrences of the same AE per study period for a subject included only once. For summaries by severity, only the worst severity for an AE will be included for a subject. Events that continue during more than one period (i.e. run-in period, Treatment Period 1, washout period, Treatment Period 2) with the same severity will only be counted once for the first study period.

The following summaries will be produced for the Treatment Period 1 and Treatment Period 2:

- All TEAEs
- All TEAEs related to auxiliary medications
- Moderate or Severe TEAEs (using maximum severity in each subject)
- Severe TEAEs (using maximum severity in each subject)

- Serious TEAEs
- Treatment-related TEAEs
- Treatment-related serious TEAEs
- TEAEs Leading to Discontinuation of Study Drug

In addition to the above summaries, the following listings by subject ID will be produced:

- All TEAEs
- Serious TEAEs
- Moderate or severe TEAEs
- TEAEs leading to discontinuation of study drug
- TEAEs leading to dose interruption
- TEAEs with a fatal outcome
- Deaths

Hepatic related toxicities will be summarized using the Drug related hepatic disorder comprehensive search Standardized MedDRA Query (Narrow) (provided in Case Retrieval Strategy (eCRS)) for Treatment Period 1 and Treatment Period 2. Hepatic related serious TEAEs will also be summarized on 1) hepatic related TEAEs leading to discontinuation of study drug or dose interruption, 2) TEAE leading to study drug discontinuation based on drug-related hepatic disorder.

AEs during run-in period is defined as AEs with start date during run-in period. All AEs will also be summarized by SOC and PT over run-in period.

AEs during washout period is defined as AEs with start date during washout period (excluding the first 30 days). The following summaries will be produced for washout period (excluding the first 30 days after the last study treatment in treatment period 1).

- All AEs
- All AEs related to auxiliary medications
- Serious AEs

Deaths will be summarized by treatment sequence and by study periods (i.e. run-in period, Treatment Period 1, washout period, Treatment Period 2).

To meet the data disclosure requirements on the public registries ClinicalTrials.gov and EudraCT, tables displaying deaths (all cause)/SAEs/Non-SAEs will be produced:

(1) Deaths and SAEs by system organ class and preferred term, on Safety set.
(2) Non-serious AEs with incidence > 10 % by system organ class and preferred term, on Safety set.

COVID-19 related infections will be summarized by MedDRA PT and study period. The following MedDRA preferred terms will be used to identify COVID-19 related infections:

- Asymptomatic COVID-19
- Coronavirus infection
- Coronavirus test positive
- COVID-19
- COVID-19 pneumonia
- Post-acute COVID-19 syndrome
- SARS-CoV-2 sepsis
- SARS-CoV-2 test positive
- SARS-CoV-2 viraemia

8.1.2 Adverse Events of Special Interest

Treatment-emergent adverse events of special interest (TEAESI) will be summarized for the Treatment Period 1 and Treatment Period 2 and will include the following categories: Fluid Retention, Anemia, Vasodilation/Hypotension, and Cardiac Failure. Proposed FDA Medical Queries (FMQs, https://www.fda.gov/drugs/development-resources/office-new-drugs-custom-medical-queries-ocmqs) will be used as the search strategies unless otherwise specified.

Table 5: Adverse Events of Special Interest

AESI	Search Criteria	Event Definition
Fluid retention	FMQ Peripheral edema (Narrow and Broad)	Any AE report identified by search criterion
Anemia	FMQ Anemia (Narrow)	Any AE report identified by search criterion
Vasodilation/Hypotension	FMQ Hypotension (Narrow)	Any AE report identified by search criterion
Cardiac Failure	FMQ Heart failure (Narrow)	Any AE report identified by search criterion

The number and percentage of TEAESIs will be summarized following the same methods used for TEAEs. The summaries to be produced include:

- All Treatment-Emergent AESIs by maximum severity
- Treatment-related Treatment-Emergent AESIs
- Treatment-related Treatment-Emergent AESIs leading to discontinuation of study drug

- Serious Treatment-Emergent AESIs

TEAESIs will be summarized alphabetically by AESI category and then within a category by descending frequency of MedDRA PT. Listings of TEAESIs by subject ID will be provided.

Adverse events of special interest (AESI) will also be summarized for the washout period (excluding the first 30 days) and will include the same categories.

8.2 Prior, Baseline, Concomitant, and Subsequent Medications

Medications will be coded using the World Health Organization (WHO) Drug Dictionary (Global B3 March 2021 version or higher) and summarized separately by Anatomical Therapeutic Chemical (ATC) Classification level 2 and preferred term for the safety analysis set. All summaries will be sorted alphabetically by ATC classification and then by decreasing total frequency of generic name within an ATC classification.

Prior, baseline, concomitant, and subsequent medications are defined as follows:

- **Prior non-study medications** are defined as medications that were stopped prior to the first dose of study treatment in Treatment Period 1, or if a subject was randomized and never received study treatment, stopped prior to the date of randomization.
- **Baseline medications** in Treatment Period 1 are defined as medications that started prior to the first dose of study treatment and ended on or after the date of the first dose of study treatment in Treatment Period 1, or if a subject was randomized and never received study treatment, started prior to the date of randomization, and ended on or after the date of randomization. In Treatment Period 2, the baseline medications are defined as medications that started prior to the first dose of study treatment in Treatment Period 2 and ended on or after the first dose of study treatment in Treatment Period 2.
- **Concomitant medications** are defined as medications that 1) started on or after the first dose of study treatment and prior to or on the last dose of study treatment in each treatment period, or 2) baseline medications that changed in dose or frequency on or after the first dose of study treatment and prior to or on the last dose of study treatment in each treatment period.
- **Washout-period medications** are defined as medications that started/changed after the last dose of study treatment in Treatment Period 1 and ended prior to the first dose of study treatment in Treatment Period 2.

Medications with completely missing start and end dates will be considered prior medications unless the medication is marked as an ongoing medication in which case it will be considered a baseline medication for both Treatment Period 1 and Treatment Period 2. Medications with partial start and end dates will be imputed as described in Table 1. Multiple occurrences of the same medication (by generic name) within a subject will be counted only once per study period in a summary. The number and percentage of prior, baseline, concomitant, and washout-period medications will be summarized by ATC class level 2 and preferred term assigned by the World Health Organization (WHO) dictionary (Global B3 March 2021 version or higher) and their usage will be summarized by treatment sequence for the safety analysis set. Baseline and

concomitant medications will also be summarized by treatment period. Subjects who report taking more than one medication will be counted only once in the total number of subjects who are taking any medication. For each specific medication, the frequency and percentage of subjects who take at least one dose of that medication will be summarized. Subjects who report a medication two or more times will be counted only once for that medication. The summaries will be sorted alphabetically by drug class and then by decreasing total frequency within a class. No statistical comparisons will be performed.

8.2.1 Concomitant Medications of Special Interest

Concomitant medications of special interest will include SGLT2 inhibitors, RAS inhibitors, diuretics, immunosuppressants including systemic corticosteroids, and blood pressure lowering agents. The number and percentage of baseline and concomitant medications of special interest will be summarized for each special interest category by ATC class level 2 and preferred term assigned by the WHO dictionary (Global B3 March 2021 version or higher) and their usage will be summarized by treatment sequence and period for the safety analysis set.

8.3 Clinical Laboratory Data

Summaries of laboratory data will be provided for the Safety analysis set and will include data collected up to the last dose of study drug plus 30 days in each treatment period. Additionally, laboratory data collected during the Washout Period will be summarized by using the baseline of Treatment Period 1. Analyses will be reported in conventional units. A by-visit figure will be provided for Hemoglobin, Brain Natriuretic Peptide, Potassium, Serum creatinine and eGFR. When values are below the LLOQ or above the ULOQ, they will be listed as such, and the closest imputed value will be used for the purpose of calculating summary statistics as specified in Section 3.4.2. Central laboratory data will be used for the summaries.

Alanine aminotransferase (ALT), aspartate aminotransferase (AST), alkaline phosphatase (ALP), total bilirubin, creatinine, potassium, and hemoglobin will be graded using the laboratory reference ranges from the central laboratory and the criteria from NCI CTCAE (Common Terminology Criteria for Adverse Events, Version 5) by the Sponsor.

Baseline, mean at each visit, and mean change from baseline at each visit in laboratory parameters will be summarized by treatment sequence and period. Summary of washout period will use the baseline of Treatment Period 1. In addition, summaries of the last value in each treatment period and the minimum and maximum values in each treatment period will be provided. Continuous variables will be summarized as described in Section 3.5.2.1 and categorical variables will be summarized as described in Section 3.5.2.2.

The number and percentage of subjects meeting the modified Hy's Law criteria will be summarized by treatment sequence and period (i.e. two treatment periods and washout period). The criteria are defined as post-baseline Total Bilirubin elevation to >=2x ULN along with concurrent ALP <2x ULN, occurring on or within 30 days after:

- A post-baseline ALT elevation to ≥ 3 x ULN
- A post-baseline AST elevation to ≥ 3 x ULN

- A post-baseline AST and/or ALT elevation to ≥ 3 x ULN

where ULN = upper limit normal. Missing ALP will be assumed to be < 2 x ULN.

The number and percentage of ALT, AST, ALP, total bilirubin, creatinine, potassium, and hemoglobin abnormalities will be summarized by grade. Hemoglobin and potassium will be graded separately for abnormally low and abnormally high abnormalities. Only the maximum CTCAE grade will be counted for subjects having the same abnormality within a time period (including scheduled and unscheduled assessments). Shift tables will be produced to summarize the shift in CTCAE grade from baseline to the maximum grade in each post-baseline study period. In addition, summaries of abnormalities by-visit and CTCAE grade will be provided. Worst post-baseline abnormalities will be summarized by study period, including both scheduled and unscheduled visits.

Summaries of the number and percentage of subjects having the following abnormalities post-baseline will be provided by treatment sequence and period (i.e. two treatment periods and washout period) for:

- ALT > 3 x ULN, > 5 x ULN, > 10 x ULN, > 20 x ULN
- AST > 3 x ULN, > 5 x ULN, > 10 x ULN, > 20 x ULN
- ALT or AST > 3 x ULN, > 5 x ULN, > 10 x ULN, > 20 x ULN
- Total Bilirubin > 1 x ULN, > 2 x ULN
- ALP > 1.5 x ULN
- ALT or AST + Total Bilirubin (based on same blood draw)
  - ALT or AST > 3 x ULN + Total Bilirubin > 1.5 x ULN
  - ALT or AST > 3 x ULN + Total Bilirubin > 2 x ULN
- ALT or AST + Total Bilirubin (based on peak values)
  - ALT or AST > 3 x ULN + Total Bilirubin > 1.5 x ULN
  - ALT or AST > 3 x ULN + Total Bilirubin > 2 x ULN

Listings for hematology, serum chemistry, and urinalysis results will be provided by subject ID number and time point [visit] in chronological order. Results falling outside of the relevant reference range will be flagged as above or below the range in the listings, as appropriate.

Spaghetti plots of liver function tests (ALP, ALT, AST, Bilirubin) over time will be produced by treatment group and subject for those subjects with a Grade 2 or higher liver function test. A scatter plot of the peak total bilirubin versus peak ALT or AST will be produced by treatment group and study period.

8.4 Body Weight and Vital Signs

The following summaries and analyses will be produced for vital signs and body weight data collected at baseline through 30 days after the last dose in each treatment period:

- Baseline, mean at each visit, and mean change from baseline at each visit will be summarized for systolic blood pressure (SBP), diastolic blood pressure (DBP), pulse rate (BPM), temperature (C°), and body weight (kg). By-visit figure will be provided. In addition, summaries of the last on-treatment value, the minimum and maximum on-treatment values, and the last post-treatment value collected in the 30-day interval after last dose of study drug will be provided.
- 3.5.2.3The number and percentage of subjects meeting any of the criteria listed below in Table will be summarized at each visit by treatment sequence and period. In addition, the number and percentage of subjects meeting any of the criteria at any time on treatment, at their last on-treatment visit and last post-treatment result collected in the 30-day interval after last dose of study drug. A by-subject listing of subjects who meet any of the criteria below in Table 6 will also be provided.

Table 6: Criteria for Potential Clinical Significance

Vital Signs	Low	High
Systolic Blood Pressure (mmHg)	≤ 100	≥ 150
Change from Baseline in Systolic Blood Pressure (mmHg)	Decrease ≥ 10	Increase ≥ 10
Diastolic Blood Pressure (mmHg)	≤ 50	≥ 100
Change from Baseline in Diastolic Blood Pressure (mmHg)	Decrease ≥ 10	Increase ≥ 5
Pulse (bpm)	≤ 50	≥ 100
Change from Baseline in Weight (kg)	NA	Increased ≥ 2.5 kg or 3% from baseline

- The number and percentage of subjects with a decrease from baseline in SBP of ≥20 mmHg and/or ≥10 mmHg in DBP will be summarized by visit within each treatment period. In addition, the number and percentage of subjects meeting the above criteria at any time on treatment, at their last on-treatment visit and last post-treatment results collected in the 30-day interval after last dose of study drug will be summarized by treatment period.

Additionally, vital signs and weight collected during the Washout Period will also be summarized.

8.5 Electrocardiograms

Electrocardiograms (ECG) for the study are collected at Screening and Day 1 in Treatment Period 1 and assessed as either 'normal', 'abnormal-not clinically significant', or 'abnormal-clinically significant' by the investigator. A listing of ECG results by subject ID and timepoint will be provided.

8.6 Other Safety Measures

None.

9 Pharmacokinetics

Atrasentan drug concentrations will be summarized at each visit by treatment sequence and period (Treatment Period 1, Treatment Period 2, and Washout Period) using descriptive statistics (n, mean, coefficient of variation, geometric mean and its coefficient of variation, standard deviation, median, first quartile, third quartile, minimum, and maximum). Per the protocol, all samples are pre-dose trough concentrations. Only concentrations for pre-dose samples collected within 18 to 30 hours (inclusive) after the previous dose will be included in the analysis.

Concentrations below the limit of quantification (BLQ) are treated as zero in summary statistics.

Pre-dose samples that were collected after dose administration at that visit should be excluded from the summary statistics.

10 Appendices

10.1 Appendix 1 Clinical Laboratory Tests

- The tests detailed below will be performed by a central laboratory except for monthly pregnancy tests and pre-screening tests.
- Additional tests may be performed at any time during the study as determined necessary by the Investigator or required by local regulations.
- Investigators must document their review of each laboratory report with an assessment of 'clinically significant' or 'not clinically significant' for any abnormal result. Clinically significant abnormal laboratory results will be recorded in the eCRF as adverse events.

**Hematology**	**Complete Chemistry**	**Urine Tests**
Hematocrit<br>Hemoglobin	Albumin<br>Alkaline phosphatase (ALP)	**Analysis of first morning void urine**:<br>Urinary concentration of protein and creatinine for

Hematology	Complete Chemistry	Urine Tests
Red Blood Cells (RBC)<br>White Blood Cells (WBC)<br>Neutrophils<br>Lymphocytes<br>Monocytes<br>Basophils<br>Eosinophils<br>Platelet count	Alanine aminotransferase (ALT) (SGPT)<br>Aspartate aminotransferase (AST) (SGOT)<br>Blood urea nitrogen (BUN)<br>Calcium<br>Potassium<br>Cholesterol (non-fasting)<br>Creatinine*<br>Direct bilirubin<br>Glucose (non-fasting)<br>Phosphorus<br>Sodium<br>Bicarbonate<br>Total bilirubin<br>Total protein<br>Uric acid<br><br>* eGFR will be calculated based on serum creatinine using the CKD-EPI equation	UPCR and UACR reporting and urinalysis (per test specification below)<br>**Analysis of 24-hour urine:**<br>• Albumin<br>• Creatinine<br>• Sodium<br>• Protein<br>• Potassium<br>• Chloride<br>• UPCR<br>• UACR<br>**Urinalysis of FMV or spot urine****<br>• Blood<br>• Glucose<br>• Ketones<br>• pH<br>• Protein<br>• Specific gravity<br>• Leukocyte esterase<br>**Reflexive microscopic analysis may be requested for certain samples<br>**Urinary Biomarker Collection**<br>**Urine pregnancy test**
		**Additional Blood Tests**<br>BNP<br>HbA1c (Screening only)<br>Serum hCG (pregnancy)
		**Additional Blood Collection**
		Blood collection for exploratory biomarkers

Hematology	Complete Chemistry	Urine Tests
		Optional blood collection for Pharmacogenetics

10.2 Appendix 2 List of Abbreviations

Term	Definition
ACE	angiotensin-converting enzyme
AE	adverse event
AESI	adverse events of special interest
ALK	alkaline phosphatase
ALT	alanine aminotransferase (SGPT)
ANCOVA	analysis of covariance
ARB	angiotensin II receptor blocker
AST	aspartate aminotransferase (SGOT)
BMI	body mass index
BNP	brain natriuretic peptide
BP	blood pressure
BUN	blood urea nitrogen
CI	confidence interval
CKD-EPI	Chronic Kidney Disease – Epidemiology Collaboration
CMH	Cochran-Mantel-Haenszel
Css	Concentration levels – steady state
ECG	Electrocardiogram
eCRF	electronic case report forms
eGFR	estimated glomerular filtration rate

**Term**	**Definition**
EoS	end of study
EoT	end of treatment
EQ-5D-5L	European Quality of Life 5 dimensions 5 levels
FMV	first morning void
Gd-IgA	Galactose deficient IgA
HbA1c	glycated hemoglobin
hCG	human chorionic gonadotropin
I/C	Intercurrent event
IDMC	Independent Data Monitoring Committee
IgAN	Immunoglobulin A nephropathy
IRT	interactive response technology
ITT	intent-to-treat
KDQOL-36	Kidney Disease Quality of Life 36 items
LLOQ	lower limit of quantification
LSMeans	Least-square means
MedDRA	Medical Dictionary for Regulatory Activities
MMRM	mixed-effects model repeated-measures
OATP	organic anion transporting polypeptides
[redacted]	
PPV	Positive predictive value
PK	Pharmacokinetics
QD	once daily
QOL	quality of life
RAS	renin-angiotensin system

Term	Definition
RBC	red blood cell (count)
SAE	serious adverse event
SAP	Statistical Analysis Plan
SGLT2	sodium glucose co-transporter 2
SGLT2i	sodium glucose co-transporter 2 inhibitor
SGOT	serum glutamic oxaloacetic transaminase (AST)
SGPT	serum glutamic pyruvic transaminase (ALT)
SMQ	Standardized MedDRA Query
SOC	system organ class
TEAE	treatment-emergent AE
UACR	urinary albumin:creatinine ratio
ULN	Upper limit of normal
ULOQ	upper limit of quantification
UPCR	urine protein:creatinine ratio
WBC	white blood cell (count)

10.3 Appendix 3 Classification Algorithm

Table 7 below outlines how prohibited/restricted medications used for analysis will be identified in the clinical database and assessed as to whether they meet the criteria for the intercurrent event, Initiation of prohibited or restricted concomitant medication.

Table 8 provides prednisone equivalent conversion factors for corticosteroids.

Table 7: Classification Algorithm

Medication Type	Search Criteria	Duration Criteria
Systemic steroids (doses > 20 mg/day of oral prednisone equivalent)	ATC2 = "CORTICOSTEROIDS FOR SYSTEMIC USE" administered orally or intravenously or intramuscularly.<br><br>Exclude ATC4 = "MINERALOCORTICOIDS"<br><br>Only include medications that are > 20 mg/day of oral prednisone equivalent.	Usage of ≥ 5 days of continuous usage<br>**OR**<br>Usage of ≥1 days in the 7 days prior to the Week 12 primary endpoint assessment<br>**OR**<br>Usage of ≥1 days in the 7 days prior to the Week 24 secondary endpoint assessment.
Immunosuppressive therapy including but not limited to mycophenolate, azathioprine, rituximab, and tacrolimus (any dose level)	ATC2 = "IMMUNOSUPPRESSANTS" for preferred terms containing: AZATHIOPRINE, CICLOSPORIN, HYDROXYCHLOROQUINE, MYCOPHENOLATE, or TACROLIMUS<br>**OR**<br>ATC2 = "ANTINEOPLASTIC AGENTS" for preferred terms containing: CYCLOPHOSPHAMIDE or RITUXIMAB	For ATC2 = "ANTINEOPLASTIC AGENTS", any usage.<br><br>The rest:<br>Usage of ≥1 days in the 7 days prior to the Week 12 primary endpoint assessment<br>**OR**<br>Usage of ≥1 days in the 7 days prior to the Week 24 secondary endpoint assessment.
Investigational or approved products for the treatment of IgAN (other than RASi and SGLT2i as they are part of the study)	Preferred terms containing: SPARSENTAN, BUDESONIDE, TELITACICEPT<br><br>Route for BUDESONIDE should be oral or intravenous.	Usage of ≥1 days in the 7 days prior to the Week 12 primary endpoint assessment<br>**OR**<br>Usage of ≥1 days in the 7 days prior to the Week 24 secondary endpoint assessment.

Table 8: Prednisone Equivalent Conversion

**Corticosteroids * (route)**	**Prednisone Equivalent Dose (mg)**	**Conversion Factor**
Prednisone (PO)	5	1
Prednisolone (PO)	5	1
Methylprednisolone (IV or PO or IM)	4	1.25
Dexamethasone (IV or PO)	0.75	6.67
Hydrocortisone (IV or PO)	20	0.25
Cortisone (PO)	25	0.20
Betamethasone (IV)	0.75	6.67
Triamcinolone (IV)	4	1.25
Deflazacort (PO)	6.7	0.75

*Preferred terms or Preferred terms containing the terms

Glucocorticoids. Facts and Comparisons 4.0. Wolters Kluwer Health, Inc. Conshohocken, PA.

Deflazacort: therapeutic index, relative potency and equivalent doses versus other corticosteroids (Parente, 2017).

11 References

Carpenter JR, Roger JH, Kenward MG (2013). Analysis of Longitudinal Trials with Protocol Deviation: A Framework for Relevant, Accessible Assumptions, and Inference via Multiple Imputation. Journal of Biopharmaceutical Statistics; 23: 1352-1371.

Dean A, Voss D. Design and Analysis of Experiments. Springer New York, NY; 1999.

de Zeeuw D, Coll B, Andress D, et al. The endothelin antagonist atrasentan lowers residual albuminuria in patients with type 2 diabetic nephropathy. J Am Soc Nephrol. 2014;25(5):1083-1093.

Heerspink HJL, Parving H-H, Andress DL, et al. Atrasentan and renal events in patients with type 2 diabetes and chronic kidney disease (SONAR): a double-blind, randomised, placebo-controlled trial. The Lancet. 2019;393(10184):1937-1947.

Inker LA, Lambers-Heerspink H, Tighiouart H, Chaudhari J, Miao S, Diva U, Mercer A, Appel

GB, Donadio JV, Floege J, Li PKT, Maes BD, Locatelli F, Praga M, Schena FP, Levey AS,

Greene T, Association of Treatment Effects on Early Change in Urine Protein and Treatment

Effects on GFR Slope in IgA Nephropathy: An Individual Participant Meta-analysis, American Journal of Kidney Diseases (2021).

Kenward MG (2015) Controlled multiple imputation methods for sensitivity analyses in longitudinal clinical trials with dropout and protocol deviation. Clin.Invest. (Lond.); 5(3):311-20.

Levey AS, Stevens LA, Schmid CH, et al. A new equation to estimate glomerular filtration rate. Ann Intern Med 2009;150: 604–12.

Lin C-W, Mostafa NM, L. Andress D, J. Brennan J, Klein CE, Awni WM. Relationship between atrasentan concentrations and urinary albumin to creatinine ratio in Western and Japanese patients with diabetic Nephropathy. Clinical Therapeutics. 2018;40(2):242-251.

Lv J, Zhang H, Wong MG, et al. Effect of Oral Methylprednisolone on Clinical Outcomes in Patients With IgA Nephropathy: The TESTING Randomized Clinical Trial. JAMA. 2017;318(5):432-442.

nQuery (2017). Sample Size and Power Calculation. “Statsols” (Statistical Solutions Ltd), Cork, Ireland.

Rauen T, Eitner F, Fitzner C, et al. Intensive Supportive Care plus Immunosuppression in IgA Nephropathy. N Engl J Med. 2015;373(23):2225-2236.

Thompson A, Carroll K, Inker LA, et al. Proteinuria reduction as a surrogate end point in trials of IgA nephropathy. Clinical Journal of the American Society of Nephrology. 2019;14(3):469-481.

Parente L. (2017): Deflazacort: therapeutic index, relative potency and equivalent doses versus other corticosteroids. BMC Pharmacology Toxicol; 18(1):1.
